# Immunoresponsive gene 1 modulates the severity of brain injury in cerebral ischaemia

**DOI:** 10.1093/braincomms/fcab187

**Published:** 2021-08-19

**Authors:** Ping-Chang Kuo, Wen-Tsan Weng, Barbara A Scofield, Destin Furnas, Hallel C Paraiso, I-Chen Yu, Jui-Hung Yen

**Affiliations:** 1 Department of Microbiology and Immunology, Indiana University School of Medicine, Fort Wayne, IN 46805, USA; 2 Department of Anatomy, Cell Biology and Physiology, Indiana University School of Medicine, Fort Wayne, IN 46805, USA

**Keywords:** IRG1, itaconate, HO-1, microglia, ischaemic stroke

## Abstract

Inflammatory stimuli induce immunoresponsive gene 1 expression that in turn catalyses the production of itaconate through diverting *cis*-aconitate away from the tricarboxylic acid cycle. The immunoregulatory effect of the immunoresponsive gene 1/itaconate axis has been recently documented in lipopolysaccharide-activated mouse and human macrophages. In addition, dimethyl itaconate, an itaconate derivative, was reported to ameliorate disease severity in the animal models of psoriasis and multiple sclerosis. Currently, whether immunoresponsive gene 1/itaconate axis exerts a modulatory effect in ischaemic stroke remains unexplored. In this study, we investigated whether immunoresponsive gene 1 plays a role in modulating ischaemic brain injury. In addition, the molecular mechanism underlying the protective effects of immunoresponsive gene 1 in ischaemic stroke was elucidated. Our results showed that immunoresponsive gene 1 was highly induced in the ischaemic brain following ischaemic injury. Interestingly, we found that *IRG1^−/−^* stroke animals exhibited exacerbated brain injury, displayed with enlarged cerebral infarct, compared to wild-type stroke controls. Furthermore, *IRG1^−/−^* stroke animals presented aggravated blood–brain barrier disruption, associated with augmented Evans blue leakage and increased immune cell infiltrates in the ischaemic brain. Moreover, *IRG1^−/−^* stroke animals displayed elevated microglia activation, demonstrated with increased CD68, CD86 and Iba1 expression. Further analysis revealed that immunoresponsive gene 1 was induced in microglia after ischaemic stroke, and deficiency in immunoresponsive gene 1 resulted in repressed microglial heme oxygenase-1 expression and exacerbated ischaemic brain injury. Notably, the administration of dimethyl itaconate to compensate for the deficiency of immunoresponsive gene 1/itaconate axis led to enhanced microglial heme oxygenase-1 expression, alleviated ischaemic brain injury, improved motor function and decreased mortality in *IRG1^−/−^* stroke animals. In summary, we demonstrate for the first time that the induction of immunoresponsive gene 1 in microglia following ischaemic stroke serves as an endogenous protective mechanism to restrain brain injury through heme oxygenase-1 up-regulation. Thus, our findings suggest that targeting immunoresponsive gene 1 may represent a novel therapeutic approach for the treatment of ischaemic stroke.

## Teaser

Kuo et al. reported that the induction of immunoresponsive gene 1 may serve as an endogenous protective mechanism to restrain brain injury following ischaemic stroke. Thus, their study suggests that targeting immunoresponsive gene 1 may represent a novel therapeutic approach for the treatment of ischaemic stroke.

## Graphical Abstract

**Graphical Abstract:**
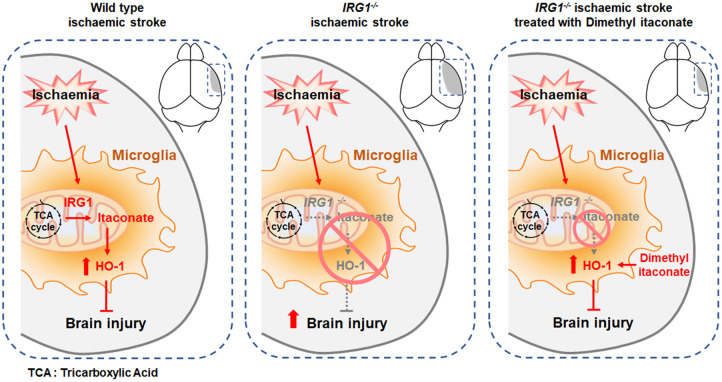

## Introduction

Stroke is a leading cause of disability in the USA and worldwide. More than 80% of stroke cases belong to ischaemic stroke, in which the occlusion of cerebral blood vessels initiates the acute phase of cerebral injury followed by excitotoxicity and oxidative damage.^1^^,^^2^ Tissue plasminogen activator is the only drug approved by the US Food and Drug Administration for the treatment of ischaemic stroke, and it functions to dissolve the blood clots that allow the re-establishment of cerebral blood flow (CBF). However, reperfusion induces reactive oxygen species generation and recruits peripheral inflammatory immune cells into the ischaemic brain, leading to enhanced neuroinflammation and exacerbated ischaemic brain injury.^3^^,^^4^

Inflammatory stimuli induce immunoresponsive gene 1 (IRG1) expression.^5–7^ IRG1 has been shown to catalyse the production of itaconate through diverting *cis*-aconitate away from the tricarboxylic acid cycle.^8^ The immunoregulatory effect of itaconate was recently documented in macrophages (MΦ). Studies showed that itaconate promoted activation of nuclear factor erythroid 2-related factor 2 (Nrf2), a key player in the antioxidant defense, in mouse and human MΦ stimulated with lipopolysaccharide (LPS).^6^^,^^7^ In addition, *IRG1*^*−*^^*/−*^ MΦ activated with LPS exhibited augmented inflammatory responses compared to wild-type (WT) MΦ stimulated with LPS.^5^ Furthermore, dimethyl itaconate (DMI), an itaconate derivative, was shown to repress IL-17-induced IκBς activation in keratinocytes and lessen disease severity in the imiquimod-induced psoriasis animal model.^9^ Moreover, DMI was shown to offer protection against myocardial and cerebral ischaemic/reperfusion injury in animal models.^10^^,^^11^ Recently, DMI was reported to attenuate neuroinflammation and ameliorate disease severity in experimental autoimmune encephalomyelitis, an animal model of multiple sclerosis, by our group.^12^

Currently, whether IRG1 exerts a protective effect against ischaemic stroke remains unexplored. Thus, we assessed whether IRG1 was induced in the ischaemic brain following middle cerebral artery occlusion (MCAO) and investigated whether IRG1 exerted protective effects on modulating brain injury in ischaemic stroke. Furthermore, we deciphered the molecular mechanism underlying the protective effects of IRG1 in ischaemic stroke. In this study, we report for the first time that the induction of IRG1 following ischaemic stroke serves as an endogenous protective mechanism to restrain ischaemic brain injury, as *IRG1*^*−*^^*/−*^ MCAO mice displayed aggravated blood–brain barrier (BBB) disruption, augmented microglia (MG) activation and exacerbated ischaemic brain injury. Mechanistic studies revealed that ischaemic stroke-induced IRG1 expression in MG that subsequently promoted microglial heme oxygenase-1 (HO-1) expression to restrain ischaemic brain injury. In summary, our findings suggest that targeting IRG1 may represent a novel therapeutic approach for the treatment of cerebral ischaemia.

## Materials and methods

### Mice

*IRG1*^*−*^^*/−*^ and its corresponding WT control C57BL/6 mice were purchased from the Jackson Laboratory (Bar Harbor, ME, USA). All animal experimental procedures were approved by the Purdue Animal Care and Use Committee and performed in strict compliance with National Institutes of Health Guidelines for the Care and Use of Laboratory Animals. All mice were housed and bred in the animal facility with controlled humidity, temperature and 12 h:12 h/light:dark cycle with free access to food and water.

### Reagents

DMI, Triphenyltetrazolium chloride (TTC), trichloroacetic acid, Evans blue and LPS (*Escherichia coli* O55: B5) were purchased from Sigma-Aldrich (St. Louis, MO, USA). 7-amino-actinomycin D viability staining solution, fixation buffer and intracellular staining permeabilization wash buffer were purchased from BioLegend (San Diego, CA, USA). Alexa Fluor 488 anti-mouse CD45 (# 103122), APC anti-mouse CD45 (# 103112), PE anti-mouse CD11b (# 101208), APC anti-mouse CD11b (# 101212), PE/Cy7 anti-mouse CD11b (# 101216), PE/Cy7 anti-mouse CD86 (# 105014), PE/Cy7 anti-mouse CD68 (# 137015) and APC anti-mouse CX3CR1 (# 149008) antibodies for flow cytometry analysis, anti-mouse MMP9 (# 819701) and anti-mouse MMP3 (# 679202) antibodies for western blots, and recombinant granulocyte-macrophage colony-stimulating factor (# 576306) and macrophage-colony stimulating factor (# 576406) for cell cultures were purchased from BioLegend (San Diego, CA, USA). Alexa Fluor 488 anti-mouse Iba1 (# ab178846) antibody for immunohistochemistry (IHC) and anti-mouse IRG1 (# ab222411) antibody for western blot analysis were purchased from Abcam (Cambridge, MA, USA). Anti-mouse HO-1 (# 10701–1-AP) antibody for IHC, western blot and flow cytometry analysis was purchased from Proteintech (Chicago, IL, USA). Alexa Fluor 546 anti-rabbit IgG secondary antibody (# A-11035) and ProLong™ Gold Antifade Mountant containing DAPI were purchased from Thermo Fisher Scientific (Waltham, MA, USA).

### Cell cultures

Primary MG was generated from P1 to P2 neonatal mice as previously described.^13^ Briefly, cerebral cortical cells from P1 to P2 neonatal mice were dissociated and plated in 75-cm^2^ culture flasks in Dulbecco’s Modified Eagle Medium/Nutrient Mixture F-12 supplemented with 10% foetal bovine serum. The medium was removed and replenished with a complete medium containing 10 ng/ml recombinant granulocyte-macrophage colony-stimulating factor on Days 4 and 8 after plating. MG was harvested for experiments by shaking the flasks at 250 rpm at 37°C for 30 min on Day 12. MΦ were generated from bone marrow cells as previously described.^14^ Briefly, bone marrow cells were cultured in Roswell Park Memorial Institute 1640 medium containing 10 ng/ml macrophage-colony stimulating factor and replenished with fresh media containing 10 ng/ml macrophage-colony stimulating factor at Day 3. MΦ were collected for experiments on Day 7.

### MCAO model

Adult male (2–4 months old) and female (2–4 and 10–12 months old) C57BL/6 and *IRG1*^*−*^^*/−*^ mice were used for cerebral ischaemia experiments as previously described.^15^ Male mice were used throughout the studies unless otherwise indicated. The transient ischaemic stroke was induced by an intraluminal suture occlusion model. Briefly, the mouse was anaesthetized by isoflurane with a mixture of 70% compressed air and 30% oxygen. During the surgical procedure, the body temperature of the animal was maintained at ∼37°C. CBF was measured before, during and after ischaemia by Laser Doppler flowmetry (moorVMS-LDF2, Moor Instruments) at the parietal bone (2 mm posterior and 3 mm lateral from Bregma). CBF was also monitored using a laser speckle contrast imaging system (moorFLPI-2 system, Moor Instruments), and the level of CBF in both hemispheres of naïve and MCAO WT and *IRG1*^*−*^^*/−*^ mice was presented using a 256-colour palette of perfusion units. The right common carotid artery was clamped by a microvascular clamp, and the right external carotid artery was exposed. A minimal incision was made in the external carotid artery stump followed by the insertion of a silicon-coated 6.0 nylon monofilament (0.21 ± 0.02 mm, Doccol Corp, Sharon, MA, USA) through the external carotid artery to the middle cerebral artery. After 40 min or 3 h of occlusion, the intraluminal suture was removed to re-establish CBF. Mice with a total CBF reduction of more than 80% were included and randomly assigned to different treatment groups. The sham surgery was conducted with the same procedures without the insertion of a suture. The investigators were blinded by the animal groups. Surgical parameters were included in Supplementary Tables 1–5.

### Infarct volume measurements

The infarct volume of the ischaemic brain was assessed by TTC staining. Briefly, the ischaemic brain harvested from MCAO mice was subjected to 2-mm coronal slicing with a rodent brain matrix followed by 1% TTC staining. After staining, the brain sections were scanned and the infarct volume was calculated by ImageJ as previously described.^15^

### Evans blue extravasation assay

Mice were intravenously administered with 4 ml/kg 2% (w/v) Evans blue dye/0.9% saline solution through the lateral tail vein. After 1 h of circulation, mice were anaesthetized and perfused with phosphate-buffered saline (PBS) to remove intravascular Evans blue. The brains were then harvested, sliced and scanned. The hemispheres of the brain were separated and homogenized with 50% trichloroacetic acid solution. Following centrifugation, the supernatants were collected and diluted with 95% ethanol in a ratio of 1:3. The level of extravascular Evans blue in the supernatant was then calculated by measuring the fluorescence with excitation at 540/25 nm and emission at 645/40 nm (BioTek Synergy HT microplate reader).

### Mononuclear cell isolation and flow cytometry analysis of extracellular and intracellular proteins

Mice subjected to MCAO were anaesthetized and perfused with PBS transcardially. After removing meninges, olfactory bulb and cerebellum, forebrains were then homogenized with 1X Hank’s balanced salt solution buffer followed by filtration through a 70-μm nylon cell strainer. After centrifugation, cells were resuspended in 30% Percoll underlayering with 70% Percoll. Following centrifugation, the mononuclear cells were then isolated from the interface between 30% and 70% Percoll. To determine MG surface expression of CD86, isolated mononuclear cells were stained with Alexa Fluor 488 anti-mouse CD45, APC anti-mouse CD11b and PE/Cy7 anti-mouse CD86. To determine MG expression of CD68 and HO-1, isolated mononuclear cells were stained with APC anti-mouse CD45 and PE anti-mouse CD11b or PE/Cy7 anti-mouse CD11b in the presence of 7-amino-actinomycin D. Following fixation and permeabilization, APC anti-mouse CD45 and PE anti-mouse CD11b-stained cells were incubated with PE/Cy7 anti-mouse CD68, or APC anti-mouse CD45 and PE/Cy7 anti-mouse CD11b-stained cells were incubated with primary anti-mouse HO-1 antibody followed by Alexa Fluor 546 secondary antibody. To determine cell infiltration, isolated mononuclear cells were stained with Alexa Fluor 488 anti-mouse CD45 and APC anti-mouse CD11b. The stained cells were then analysed by flow cytometer (BD FACSVerse).

### Quantitative polymerase chain reaction (Q-PCR)

The expression of *Irg1, Ho-1, Cx3cr1, Gfap* *and CD31* was measured by Q-PCR as previously described.^16^ The primers used were *Irg1*: sense 5′-GCAACATGATGCTCAAGTCTG-3′ and antisense 5′-TGCTCCTCCGAATGATACCA-3′; *Ho-1*: sense 5′-GCTGGTGATGGCTTCCTTG T-3’ and antisense 5′-ACTGGGTTCTGCTTGTTGCG-3′; *Cx3cr1*: sense 5′-CAGCATCGACCG GTACCTT-3′ and antisense 5′-GCTGCACTGTCCGGTTGTT-3′; *Gfap*: sense 5′-AGAAAGGT TGAATCGCTGGA-3′ and antisense 5′-CGGCGATAGTCGTTAGCTTC-3′; *CD31*: sense 5′-CCAAAGCCAGTAGCATCATGGTC-3′ and antisense 5′-GGATGGTGAAGTTGGCTACAG G-3′.

### Western blot analysis

Protein samples were prepared in radioimmunoprecipitation assay buffer [50 mM Tris-HCl (pH 8.0), 150 mM NaCl, 1% NP-40, 0.5% sodium deoxycholate, 1 mM phenylmethylsulfonyl fluoride and 1X protease inhibitor cocktail] with 0.3% SDS (brain tissue) or 0.1% SDS (cultured cell). The protein concentrations were detected by Pierce™ BCA Protein Assay Kit (Thermo Fisher Scientific, Waltham, MA, USA). Protein electrophoresis was conducted on 10% SDS-PAGE. After being transferred to polyvinylidene difluoride membranes (Millipore Temecula, CA, USA), the blots were reacted with specific antibodies and detected by using Immobilon Western Chemiluminescent HRP Substrate (Millipore, Temecula, CA, USA).

### IHC

Brain samples were dissected and fixed with 4% paraformaldehyde in PBS at 4°C overnight. After 6% and 30% sucrose dehydration, brain samples were embedded in the optimal cutting temperature compound and cut into 40 μm cryosections. Cryosections were air-dried at room temperature (RT) and permeabilized in PBS containing 0.5% Triton X-100 for 30 min. Following blocking with goat serum (5% goat serum, 0.25% Triton X-100 in PBS) at RT for 1 h, sections were incubated with primary anti-mouse HO-1 antibody overnight at 4°C. After wash, sections were stained with Alexa Fluor 546 anti-rabbit IgG secondary antibody for 2 h at RT. Following the wash, sections were then stained with Alexa Fluor 488 anti-mouse Iba1 antibody for 2 h at RT. Negative controls were prepared by incubating sections with secondary antibody for 2 h followed by staining with Alexa Fluor 488 anti-mouse IgG antibody for 2 h at RT. Following the wash, all samples were coverslipped with ProLong™ Gold Antifade Mountant containing DAPI. The Z-stack images of two random spots on cortex and striatum of peri-infarct regions at the position around 0.8 mm away from the bregma were taken from one brain section per animal by confocal microscopy (Fluoview FV10i, Olympus) to visualize Iba1 and HO-1 expression and co-localization. The number of Iba1^+^ cells and HO-1^+^Iba-1^+^ cells per mm^2^, and the intensity of HO-1 expression were quantified by ImageJ.

### CD11b isolation of MG from brain

CD11b^+^ cell isolation was performed by using the EasySep™ Mouse CD11b Positive Selection Kit II (STEMCELL Technologies, MA). The flowchart of column-free magnetic separation is shown in Supplementary Fig. 4. In brief, the brains were harvested and separated into contralateral and ipsilateral hemispheres. Two pooled contralateral or ipsilateral hemispheres were gently triturated and then passed through a 70-μm nylon cell strainer. Cells were then centrifuged and resuspended in 30% Percoll solution. After centrifugation, myelin debris was removed, and cells were resuspended and then incubated with CD11b magnetic beads. Following magnetic separation, the CD11b^+^ and CD11b^−^ cells were collected and subjected to further analyses.

### Rotarod test

Motor co-ordination was assessed by using a rotarod test (Model 47600, Ugo Basile, Varese, Italy). During the training sessions, mice were placed on a rotating drum set to accelerate from 4 to 80 rpm over a 5-min period for three trials with 30-min intervals every day for 3 consecutive days. During the testing sessions, mice were given three trials on the rotarod apparatus set to accelerate from 4 to 80 rpm with a maximum time of 300 s and 30 min intertrial rest interval at Day 2, 4 and 6 post-injury. The latency to fall was recorded as a measure of motor function.

### Statistical analysis

All results are given as mean ± SEM. The sample size was determined to be adequate based on our previous studies and also prior literature using similar experimental paradigms. The collection of data was randomized for all experiments. The normal distribution of the data in each group was confirmed by Shapiro–Wilk normality test. Comparisons between two groups and one variable were performed by a two-tailed unpaired *t*-test, whereas comparisons among multiple groups were performed by one-way ANOVA (one variable) or two-way ANOVA (two variables) followed by Tukey *post hoc* test. For samples that did not pass the normality test, comparisons between two groups and one variable were performed by Mann–Whitney U test. Statistical analyses were performed using GraphPad Prism 9 software. Statistical significance was determined as *P* < 0.05.

### Data availability

All data and detailed experimental procedures of this study are available from the corresponding author upon reasonable request. Original uncropped western blot images can be found in Supplementary Fig. 6.

## Results

### Ischaemic stroke induces IRG1 expression in the ischaemic brain; however, deficiency in IRG1 results in exacerbated ischaemic brain injury

**Figure 1:**
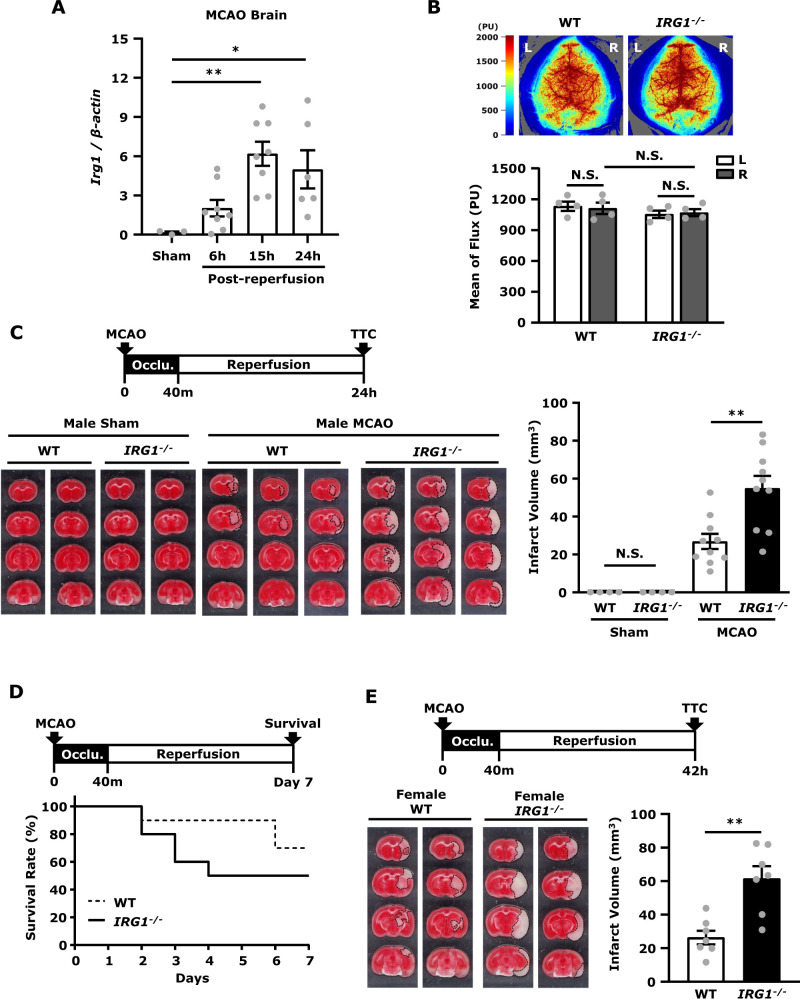
**Ischaemic stroke induces IRG1 expression in the ischaemic brain, however deficiency in IRG1 results in exacerbated ischaemic brain injury.** (**A**) C57BL/6 mice were subjected to sham or 40 min MCAO. The ipsilateral hemispheres of sham controls were harvested at 15 h post-reperfusion (*n* = 3 mice), and the ipsilateral hemispheres of MCAO mice were harvested at 6, 15 and 24 h post-reperfusion (*n* = 6–8 mice per time point). Samples were then subjected to Q-PCR analysis for IRG1 expression. *P < 0.05, **P < 0.01 by one-way ANOVA. (**B**) Naïve WT and *IRG1*^−^^*/*^^−^ mice were subjected to the measurement of CBF in right (R) and left (L) hemispheres. The representative CBF images of WT and *IRG1*^−^^*/*^^−^ are shown, and the perfusion units (PU) in both hemispheres of WT and *IRG1*^−^^*/*^^−^ mice were measured and quantified (*n* = 4 mice per group). N.S., no significant differences by two-way ANOVA. (**C**) WT and *IRG1*^−^^*/*^^−^ mice were subjected to sham (*n* = 4 mice per group) and MCAO (*n* = 10 mice per group) surgery. Twenty-four hours after injury, mice were sacrificed, and the ischaemic brains were harvested and sliced (2 mm) followed by TTC staining. The representative TTC-stained samples of WT and *IRG1*^−^^*/*^^−^ sham (*n* = 2 mice per group) and MCAO mice (*n* = 3 mice per group) are shown. The infarct volumes were also measured. **P < 0.01, N.S., no significant differences by one-way ANOVA. (**D**) The Kaplan-Meier survival curve of WT and *IRG1*^−^^*/*^^−^ MCAO mice (*n* = 10 mice per group) was evaluated up to Day 7 post-injury. (**E**) Middle-aged (10–12 months old) female WT and *IRG1*^−^^*/*^^−^ mice were subjected to MCAO (*n* = 7 mice per group). Forty-two hours post-injury, mice were sacrificed, and the ischaemic brains were harvested and sliced (2 mm) followed by TTC staining. Two representative TTC-stained samples of WT and *IRG1*^−^^*/*^^−^ MCAO mice are shown. The infarct volumes were also measured. **P < 0.01 by unpaired *t*-test.

To first evaluate whether ischaemic stroke induces IRG1 expression, C57BL/6 mice were subjected to sham and MCAO, and the ipsilateral hemispheres of ischaemic brains were harvested at 6, 15 and 24 h post-reperfusion followed by Q-PCR analysis to assess IRG1 expression. Our results showed that ischaemic stroke-induced IRG1 expression in the ischaemic brain at 6 h post-reperfusion and the induction of IRG1 reached a peak at 15 h post-reperfusion (Fig. 1A). To further investigate whether ischaemia-induced IRG1 expression played a role in ischaemic stroke, we compared the level of ischaemic brain injury between *IRG1*^*−*^^*/−*^ MCAO mice and its corresponding WT MCAO controls. We first evaluated whether naïve WT and *IRG1*^*−*^^*/−*^ mice exhibited a comparable level of CBF reading and BBB integrity because these parameters would affect the outcome of ischaemic brain injury. The laser speckle imaging system was used to measure CBF, and we observed a similar level of CBF reading between naïve WT and *IRG1*^*−*^^*/−*^ mice (Fig. 1B). Naïve WT and *IRG1*^*−*^^*/−*^ mice were then subjected to Evans blue administration to assess whether IRG1 deficiency compromised BBB integrity. Similar to naïve WT mice, no Evans blue leakage was detected in the brain of naïve *IRG1*^*−*^^*/−*^ mice (Supplementary Fig. 1), suggesting IRG1 deficiency does not alter BBB integrity under normal conditions.

We then subjected WT and *IRG1*^*−*^^*/−*^ mice to sham and MCAO surgery, and the level of ischaemic brain injury was compared between WT and *IRG1*^*−*^^*/−*^ MCAO mice. WT and *IRG1*^*−*^^*/−*^ mice subjected to sham surgery did not develop any detectable cerebral infarct. In contrast, WT and *IRG1*^*−*^^*/−*^ mice subjected to MCAO surgery developed cerebral infarct (Fig. 1C). Although the physiological parameters of WT and *IRG1*^*−*^^*/−*^ mice during MCAO were comparable (Supplementary Table 1), *IRG1*^*−*^^*/−*^ MCAO mice developed a much severe brain injury compared to WT MCAO controls (Fig. 1C). In WT MCAO mice, we observed the infarct was mainly developed in the striatum of the ischaemic brain, whereas, in *IRG1*^*−*^^*/−*^ MCAO mice, the infarct was further extended to the cortex of the ischaemic brain at Day 1 post-injury (Fig. 1C). The analysis of ischaemic brain infarct revealed that *IRG1*^*−*^^*/−*^ MCAO mice had a larger infarct than WT MCAO mice (*IRG1*^*−*^^*/−*^ 55.0 ± 6.5 mm^3^ versus WT 26.9 ± 4.0 mm^3^) (Fig. 1C). In addition, the long-term survival of WT and *IRG1*^*−*^^*/−*^ MCAO mice was assessed. We observed *IRG1*^*−*^^*/−*^ MCAO mice exhibited a decreased survival compared to WT MCAO mice. Our results showed that *IRG1*^*−*^^*/−*^ MCAO mice had a survival rate of 50%, whereas WT MCAO mice had a survival rate of 70% by Day 7 post-injury (Fig. 1D). Finally, female WT and *IRG1*^*−*^^*/−*^ mice were subjected to MCAO to assess the level of ischaemic brain injury. Since female mice develop a less severe brain injury compared to male mice in ischaemic stroke,^17^^,^^18^ the ischaemic brain injury was assessed in middle-aged female WT and *IRG1*^*−*^^*/−*^ MCAO mice at Day 2 post-injury. Consistently, although the physiological parameters of female WT and *IRG1*^*−*^^*/−*^ mice during MCAO were comparable (Supplementary Table 2), female *IRG1*^*−*^^*/−*^ MCAO mice developed a larger infarct than female WT MCAO mice (*IRG1*^*−*^^*/−*^ 61.5 ± 7.4 mm^3^ versus WT 26.4 ± 4.1 mm^3^) (Fig. 1E). Taken altogether, our results demonstrate that ischaemic stroke induces IRG1 expression in the ischaemic brain; however, deficiency in IRG1 results in exacerbated brain injury after ischaemia stroke.

### IRG1 deficiency exacerbates BBB disruption in ischaemic stroke

**Figure 2:**
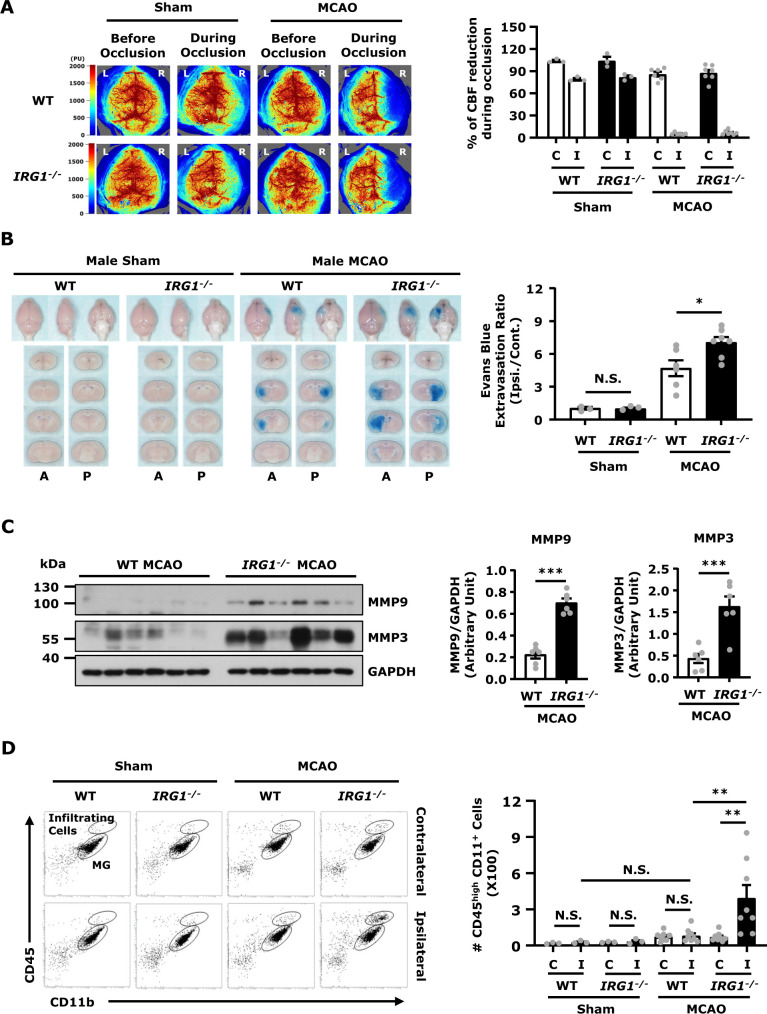
**IRG1 deficiency exacerbates BBB disruption in ischaemic stroke.** (**A**) WT and *IRG1*^−^^*/*^^−^ mice were subjected to the measurement of CBF before and during sham (*n* = 3 mice per group) and MCAO surgeries (*n* = 6–7 mice per group). The representative CBF images of WT and *IRG1*^−^^*/*^^−^ sham and MCAO mice are shown, and the percentage of CBF reduction (CBF reading during occlusion/CBF reading before occlusion) in the contralateral (C) and ipsilateral (I) hemispheres were calculated. (**B**) WT and *IRG1*^−^^*/*^^−^ mice were subjected to sham (*n* = 3 mice per group) or 3 h MCAO (*n* = 6–7 mice per group) followed by 3.5 h reperfusion. One hour before scarification, mice were intravenously injected with Evans blue. At 6.5 h post-injury, the ischaemic brains were then harvested and subjected to sectioning and imaging, and the Evans blue leakage in the contralateral and ipsilateral hemispheres was then quantified. The representative ischaemic brain images of WT and *IRG1*^−^^*/*^^−^ sham and MCAO mice are shown, and the Evans blue extravasation ratio of ipsilateral (Ipsi.) hemisphere/contralateral (Cont.) hemisphere was determined. A: anterior surface; P: posterior surface. *P < 0.05, N.S., no significant differences by one-way ANOVA. (**C**) WT and *IRG1*^−^^*/*^^−^ mice were subjected to 3 h MCAO followed by 3.5 h reperfusion (*n* = 6 mice per group). The ipsilateral hemispheres of ischaemic brains were then harvested and subjected to western blot analysis for MMP9 and MMP3 expression. The level of MMP9 and MMP3 expression was also quantified. ***P < 0.001 by unpaired *t*-test. (**D**) WT and *IRG1*^−^^*/*^^−^ mice were subjected to sham or 40 min MCAO surgery. Twenty-four hours post-injury, mononuclear cells were isolated from the contralateral (C) and ipsilateral (I) hemispheres of WT and *IRG1*^−^^*/*^^−^ sham (*n* = 3 mice per group) and MCAO mice (*n* = 8 mice per group). The isolated cells were then stained with antibodies against CD45 and CD11b followed by flow cytometry analysis. The CNS infiltrating immune cells were determined based on their high expression of CD45 and positive expression of CD11b (CD45^hi^CD11b^+^). **P < 0.01, N.S., no significant differences by two-way ANOVA.

To determine whether IRG1 deficiency affects BBB integrity after ischaemic stroke, WT and *IRG1*^*−*^^*/−*^ mice were subjected to sham or MCAO to assess the level of BBB disruption. We subjected mice to 3 h occlusion followed by 3.5 h reperfusion, according to our previously established conditions,^19^ to assess the level of Evans blue leakage in the ischaemic brain. Our results showed that there was no Evans blue leakage in both WT and *IRG1*^*−*^^*/−*^ sham animals (Fig. 2B). In contrast, although WT and *IRG1*^*−*^^*/−*^ mice showed a comparable level of CBF reduction during cerebral occlusion (Fig. 2A), *IRG1*^*−*^^*/−*^ MCAO mice displayed a significant increase of Evans blue leakage in the ischaemic brain compared to WT MCAO mice (Fig. 2B), indicating there was a higher level of BBB disruption in *IRG1*^*−*^^*/−*^ MCAO mice than WT MCAO mice. In addition, female WT and *IRG1*^*−*^^*/−*^ MCAO mice were subjected to Evans blue administration to assess the level of BBB disruption. Consistently, we observed female *IRG1*^*−*^^*/−*^ MCAO mice displayed a higher level of Evans blue leakage than female WT MCAO mice (Supplementary Fig. 2A). Since matrix metalloproteinases, MMP9 and MMP3, were previously reported to exert detrimental effects on the disruption of BBB,^20^ we explored whether aggravated BBB disruption in *IRG1*^*−*^^*/−*^ MCAO mice was due to increased production of MMP9 and MMP3 in the ischaemic brain. Indeed, a significant increase of MMP9 and MMP3 expression was detected in the ischaemic brain of *IRG1*^*−*^^*/−*^ MCAO mice compared to that of WT MCAO controls (Fig. 2C), suggesting IRG1 deficiency results in dysregulated production of MMP9 and MMP3 in the ischaemic brain that may, in turn, exacerbate BBB disruption.

We then assessed whether aggravated BBB disruption in *IRG1*^*−*^^*/−*^ MCAO mice promoted immune cell infiltration of the ischaemic brain. WT and *IRG1*^*−*^^*/−*^ mice were subjected to sham or 40 min MCAO. Mononuclear cells were isolated from the ischaemic brain of WT and *IRG1*^*−*^^*/−*^ sham and MCAO mice at 24 h post-injury, and the isolated cells were then stained with CD45 and CD11b antibodies followed by flow cytometry analysis. The CNS infiltrating immune cells were determined based on their high expression of CD45 (CD45^hi^) and positive expression of CD11b (CD11b^+^). Our results showed that there was no significant increase of CD45^hi^CD11b^+^ cells in the ipsilateral hemisphere compared to the contralateral hemisphere in WT MCAO mice at Day 1 post-injury. In contrast, a significant increase of CD45^hi^CD11b^+^ cells was observed in the ipsilateral hemisphere of *IRG1*^*−*^^*/−*^ MCAO mice compared to the ipsilateral hemisphere of WT MCAO mice or the contralateral hemisphere of *IRG1*^*−*^^*/−*^ MCAO mice at Day 1 post-stroke (Fig. 2D). Collectively, these results demonstrate that IRG1 deficiency exacerbates BBB disruption that may subsequently promote the peripheral immune cell infiltration of the CNS after ischaemic stroke.

### IRG1 deficiency promotes MG activation in ischaemic stroke

**Figure 3:**
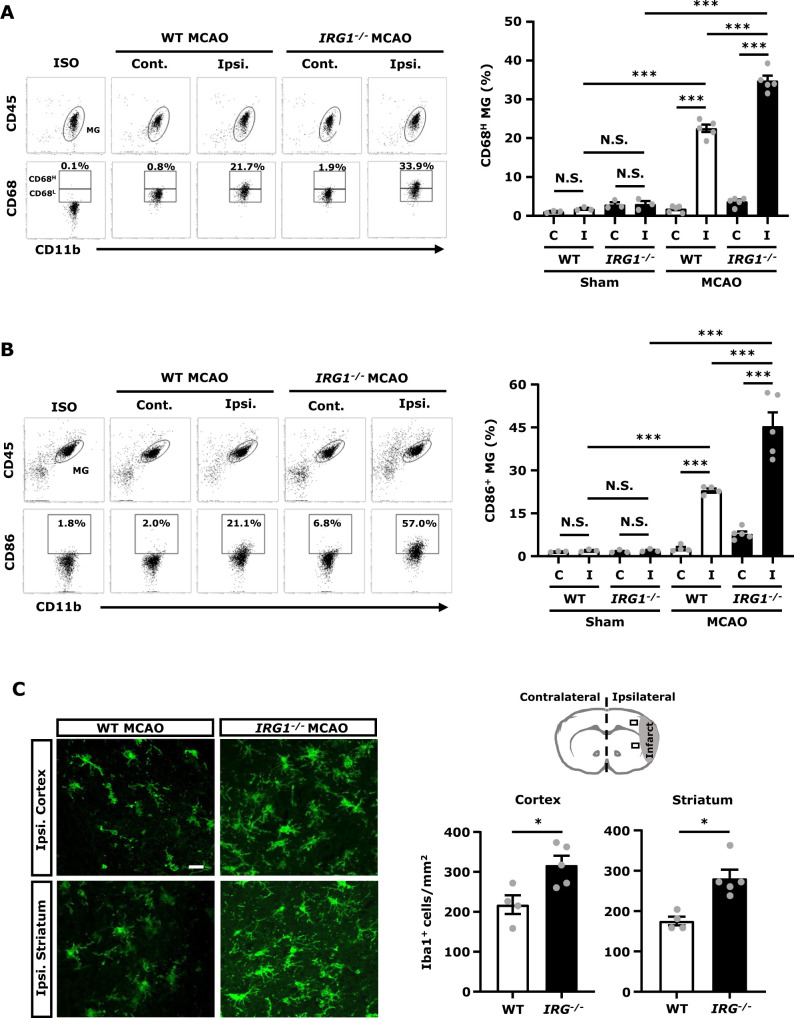
**IRG1 deficiency promotes MG activation in ischaemic stroke.** WT and *IRG1*^−^^*/*^^−^ mice were subjected to sham or 40 min MCAO surgery. (**A**) At 15 h post-injury, the ischaemic brains were harvested from WT and *IRG1*^−^^*/*^^−^ sham (*n* = 3 mice per group) and MCAO mice (*n* = 5 mice per group), and separated into the contralateral (Cont.; C) and ipsilateral (Ipsi.; I) hemispheres followed by mononuclear cell isolation. The isolated cells were stained with antibodies of CD45 and CD11b in the presence of 7-amino-actinomycin D and then stained intracellularly with CD68 followed by flow cytometry analysis. MG was determined based on their expression of CD45^int^CD11b^+^, and isotype controls (ISO) were used as a negative control to determine MG positive for CD68 expression. The gating of CD68 low (CD68^L^) was based on the basal expression of CD68 in MG in WT sham controls, and the expression level of CD68 higher than CD68^L^ was then determined as CD68 high (CD68^H^). The percentage of CD68^H^ MG was then measured. ***P < 0.001, N.S., no significant differences by two-way ANOVA. (**B**) At 15 h post-injury, mononuclear cells isolated from the contralateral (Cont.; C) and ipsilateral (Ipsi.; I) hemispheres of WT and *IRG1*^−^^*/*^^−^ sham (*n* = 3 mice per group) and MCAO mice (*n* = 4–5 mice per group) were stained with antibodies of CD45, CD11b and CD86 followed by flow cytometry analysis. ISO was used as a negative control to determine MG positive for CD86 expression. ***P < 0.001, N.S., no significant differences by two-way ANOVA. (**C**) At 24 h post-injury, the ischaemic brains were harvested from WT and *IRG1*^−^^*/*^^−^ MCAO mice followed by IHC analysis to determine Iba1 expression (*n* = 4–5 mice per group). The representative images of Iba1 immunostaining in the ipsilateral cortex and striatum of peri-infarct regions of WT and *IRG1*^−^^*/*^^−^ MCAO mice are shown. Iba1^+^ cells per mm^2^ were also quantified. Scale bar: 20 µm. *P < 0.05 by unpaired *t*-test.

To determine whether exacerbated ischaemic brain injury in *IRG1*^*−*^^*/−*^ MCAO mice was due to elevated neuroinflammation, the level of MG activation in WT and *IRG1*^*−*^^*/−*^ MCAO mice was assessed. The ischaemic brains were harvested from WT and *IRG1*^*−*^^*/−*^ MCAO mice followed by mononuclear cell isolation, and the isolated cells were subjected to CD45, CD11b and CD68 staining followed by flow cytometry analysis to assess the expression of CD68, an activation marker, in MG. MG was determined based on their intermediate expression of CD45 (CD45^int^) and positive expression of CD11b (CD11b^+^). Our results showed that a low level of CD68 expression (CD68^L^) in MG was observed in sham controls as well as in the contralateral hemisphere of WT and *IRG1*^*−*^^*/−*^ MCAO mice, indicating MG express a basal level of CD68 (Supplementary Figs 3A and B and Fig. 3A). Notably, cerebral ischaemia enhanced CD68 expression (CD68^H^) in MG, and our results showed that the frequency of CD68^H^ MG was significantly higher in the ipsilateral hemisphere of *IRG1*^*−*^^*/−*^ MCAO mice than that of WT MCAO mice (Fig. 3A). In addition, MG expression of CD86, a maturation marker, was measured to confirm the level of MG activation in WT and *IRG1*^*−*^^*/−*^ MCAO mice. Our results showed that cerebral ischaemia increased the frequency of CD86^+^ MG in the ipsilateral hemisphere of WT and *IRG1*^*−*^^*/−*^ MCAO mice compared to the ipsilateral hemisphere of WT and *IRG1*^*−*^^*/−*^ sham controls, or the contralateral hemisphere of WT and *IRG1*^*−*^^*/−*^ MCAO mice (Fig. 3B and Supplementary Fig. 3C). Consistent with the results of CD68^H^ expression in MG, the frequency of CD86^+^ MG was also significantly higher in *IRG1*^*−*^^*/−*^ MCAO mice than WT MCAO mice (Fig. 3B). Finally, ischaemic brain tissues were subjected to IHC to assess the level of Iba1^+^ cells in WT and *IRG1*^*−*^^*/−*^ MCAO mice. We found that the number of Iba1^+^ cells was significantly higher in the ipsilateral cortex and striatum of *IRG1*^*−*^^*/−*^ MCAO mice than those of WT MCAO mice (Fig. 3C). Taken altogether, these results demonstrate that *IRG1*^*−*^^*/−*^ MCAO mice exhibit elevated MG activation, displayed with increased CD68^H^ and CD86^+^ MG as well as Iba1^+^ cells in the ischaemic brain, compared to WT MCAO mice, suggesting IRG1 deficiency aggravates MG activation in ischaemic stroke.

### IRG1 deficiency represses HO-1 expression in the ischaemic brain

**Figure 4:**
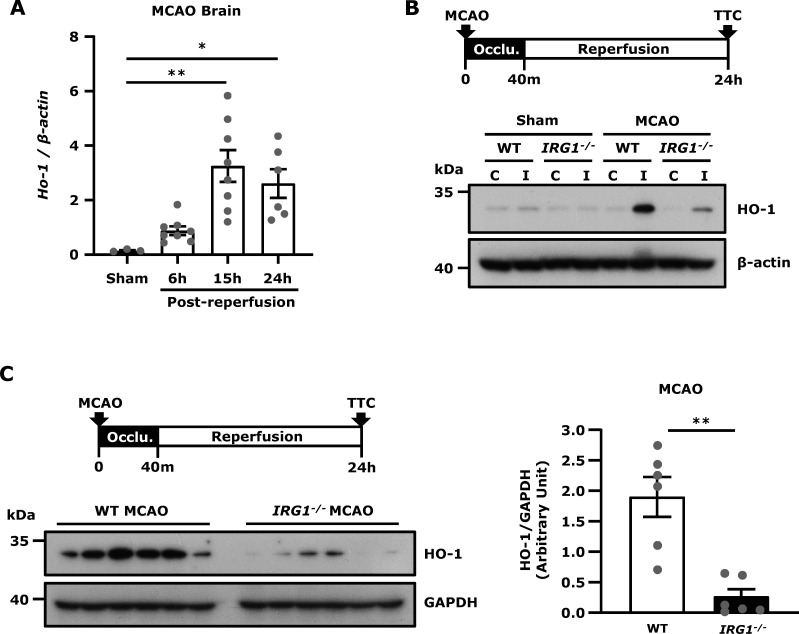
**IRG1 deficiency represses HO-1 expression in the ischaemic brain.** (**A**) WT mice were subjected to sham or 40 min MCAO. The ipsilateral hemispheres of sham controls were harvested at 15 h post-reperfusion (*n* = 3 mice), and the ipsilateral hemispheres of MCAO mice were harvested at 6, 15 and 24 h post-reperfusion (*n* = 6–8 mice per time point). Samples were then subjected to Q-PCR analysis for HO-1 expression. *P < 0.05, **P < 0.01 by one-way ANOVA. (**B**) WT and *IRG1*^−^^*/*^^−^ mice were subjected to sham or 40 min MCAO. At 24 h post-injury, the contralateral and ipsilateral hemisphere tissues were harvested and subjected to western blot analysis for HO-1 expression. The representative results of HO-1 expression in the contralateral (C) and ipsilateral (I) hemispheres of sham and MCAO mice are shown. Similar results were observed in three independent experiments. (**C**) The ischaemic brains were harvested from WT and *IRG1*^−^^*/*^^−^ MCAO mice at 24 h post-injury, and the ipsilateral hemisphere tissues were then subjected to western blot analysis for HO-1 expression. The level of HO-1 expression was also quantified (*n* = 6 mice per group). **P < 0.01 by Mann–Whitney U test.

IRG1 induces itaconate production, and itaconate has been shown to induce Nrf2 activation and subsequent HO-1 up-regulation.^5^^,^^8^^,^^9^ In addition, the induction of HO-1 has been reported to exert protection against ischaemic brain injury.^15^^,^^21^^,^^22^ Thus, we investigated whether ischaemic stroke-induced endogenous HO-1 expression in the ischaemic brain. We observed the pattern of HO-1 mRNA expression was similar to IRG1 mRNA expression in the ischaemic brain in which HO-1 expression was upregulated at 6 h post-reperfusion and reached a peak at 15 h post-reperfusion (Fig. 4A). In addition, we found that HO-1 protein expression was only upregulated in the ipsilateral hemisphere of MCAO mice but not in the contralateral hemisphere of MCAO mice nor both hemispheres of sham controls at 24 h post-injury (Fig. 4B). We then compared the level of HO-1 expression in the ischaemic brain of WT and *IRG1*^*−*^^*/−*^ MCAO mice and observed that HO-1 expression was significantly repressed in the ischaemic brain of *IRG1*^*−*^^*/−*^ MCAO mice compared to that in WT MCAO mice (Fig. 4C). Collectively, our results demonstrate that ischaemic stroke induces HO-1 expression, however deficiency in IRG1 results in repressed HO-1 expression in the ischaemic brain.

### IRG1 is mainly expressed in MG, and deficiency in IRG1 results in repressed HO-1 expression in MG *in vivo and in vitro*

**Figure 5:**
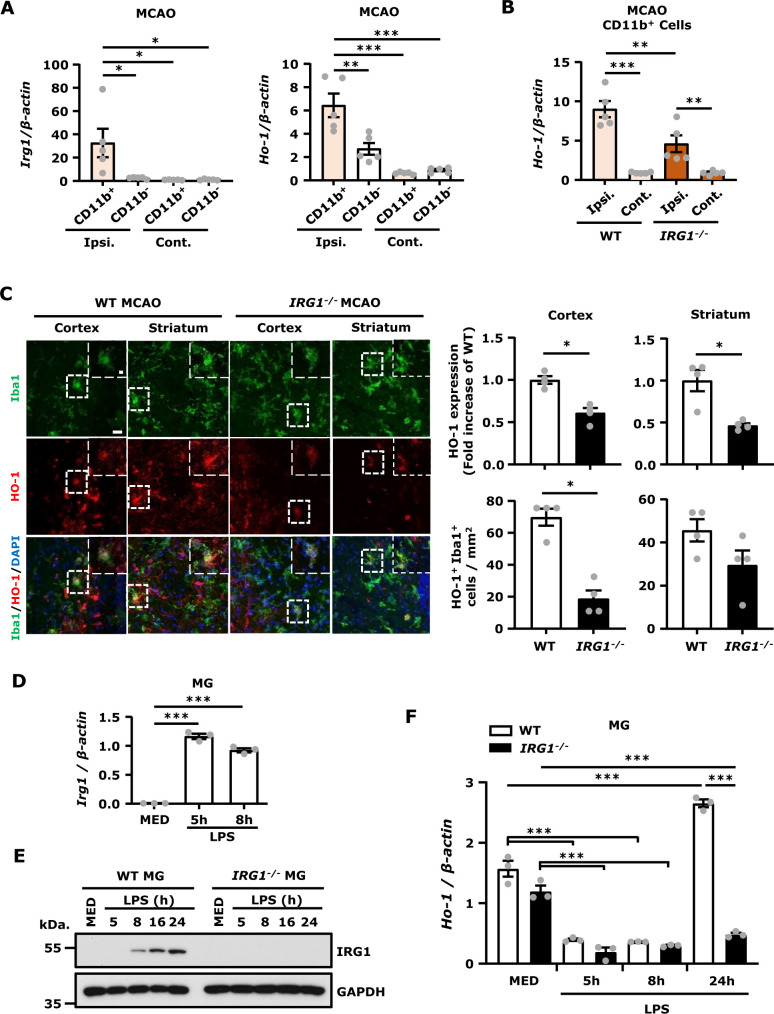
**IRG1 is mainly expressed in MG, and deficiency in IRG1 results in repressed HO-1 expression in MG *in vivo* and *in vitro*.** (**A**) C57BL/6 mice were subjected to 40 min MCAO. The ipsilateral (Ipsi.) and contralateral (Cont.) hemispheres were harvested at 6 h post-reperfusion. Two ipsilateral or contralateral hemispheres were pooled and subjected to CD11b isolation. The CD11b^+^ and CD11b^−^ cells were then collected for RNA extraction followed by Q-PCR analysis for IRG1 and HO-1 expression (*n* = 5 per group, each *n* represents pooled two brain samples). *P < 0.05, **P < 0.01, ***P < 0.001 by two-way ANOVA. (**B**) WT and *IRG1*^−^^*/*^^−^ mice were subjected to 40 min MCAO. Two ipsilateral or contralateral hemispheres harvested from WT and *IRG1*^−^^*/*^^−^ MCAO mice at 24 h post-injury were pooled and subjected to CD11b isolation. The CD11b^+^ cells were then collected for RNA extraction followed by Q-PCR analysis for HO-1 expression (*n* = 5 per group, each *n* represents pooled two brain samples). **P < 0.01, ***P < 0.001 by two-way ANOVA. (**C**) The ischaemic brains of WT and *IRG1*^−^^*/*^^−^ MCAO mice harvested at 24 h post-injury were subjected to IHC analysis to determine Iba1 and HO-1 expression (*n* = 4 mice per group). The representative images of HO-1 and Iba1 immunoreactivity in the ipsilateral cortex and striatum of peri-infarct regions are shown. The level of HO-1 immunoreactivity and the number of HO-1^+^ Iba1^+^ cells were quantified. Scale bar: 20 µm; 5 μm in magnified boxes. *P < 0.05 by unpaired *t*-test. (**D**) Primary MG generated from WT mice were stimulated with LPS 100 ng/ml for 5 or 8 h, and cells were then harvested and subjected to Q-PCR analysis for IRG1 expression (*n* = 3 technique replicates per group). ***P < 0.001 by one-way ANOVA. (**E**) Primary MG generated from WT and *IRG1*^−^^*/*^^−^ mice were stimulated with LPS 100 ng/ml for a time course followed by western blot analysis for IRG1 expression. (**F**) Primary WT and *IRG1*^−^^*/*^^−^ MG were treated with LPS 100 ng/ml for a time course, and cells were then harvested followed by Q-PCR analysis for HO-1 expression (*n* = 3 technique replicates per group). ***P < 0.001 by two-way ANOVA. The results of **D–F** represent 3–4 independent biological replicates.

IRG1 is a mitochondrial enzyme that catalyses the production of itaconate under inflammatory conditions, principally in cells of the myeloid lineage.^23^ To investigate whether MG, myeloid lineage cells, represent the main producer of IRG1 in the ischaemic brain, we conducted CD11b isolations from the contralateral and ipsilateral hemispheres and subjected isolated CD11b^+^ and CD11b^−^ cells to Q-PCR analysis to assess gene expression. We verified the purity of MG in isolated CD11b^+^ cells by flow cytometry analysis, and our results showed that more than 85% of CD11b^+^ cells are CX3CR1^+^ MG (Supplementary Fig. 4A). In addition, we assessed signature genes of MG, astrocytes and brain endothelial cells, such as CX3CR1, GFAP and CD31, respectively, in CD11b^+^ and CD11b^−^ cells isolated from the contralateral and ipsilateral hemispheres of MCAO mice. Our results showed that CX3CR1 expression was only detectable in CD11b^+^ cells but not in CD11b^−^ cells, whereas GFAP and CD31 expression were largely detected in CD11b^−^ cells with a lesser degree in CD11b^+^ cells (Supplementary Fig. 4B), suggesting that the CD11b^+^ cells contain mostly MG and the CD11b^−^ cells contain non-MG, such as astrocytes and brain endothelial cells. We then compared IRG1 expression in the CD11b^+^ and CD11b^−^ cells, and our results showed that IRG1 was only highly upregulated in CD11b^+^ cells isolated from the ipsilateral hemisphere but not in the CD11b^−^ cells isolated from the ipsilateral hemisphere nor in the CD11b^+^ or CD11b^−^ cells isolated from the contralateral hemisphere of MCAO mice (Fig. 5A, left panel), indicating IRG1 is only upregulated in MG in the injured hemisphere after ischaemic stroke. Furthermore, we assessed HO-1 expression in the CD11b^+^ and CD11b^−^ cells and found that HO-1 was highly upregulated in the CD11b^+^ cells but only slightly upregulated in the CD11b^−^ cells isolated from the ipsilateral hemisphere compared to the CD11b^+^ and CD11b^−^ cells isolated from the contralateral hemisphere (Fig. 5A right panel), suggesting HO-1 is highly expressed in MG in the ischaemic brain. Finally, we compared the level of HO-1 expression in the CD11b^+^ cells isolated from WT and *IRG1*^−^^*/*^^−^ MCAO mice to assess whether IRG1 promoted HO-1 expression in MG. Indeed, our results showed that the expression of HO-1 was significantly higher in the CD11b^+^ cells isolated from WT MCAO mice than those isolated from *IRG1*^−^^*/*^^−^ MCAO mice (Fig. 5B). To confirm the observed mRNA results at the protein levels, the ischaemic brains were harvested from WT and *IRG1*^−^^*/*^^−^ MCAO mice and then subjected to IHC to measure HO-1 and Iba1 immunoactivity. The co-localization of Iba1 and HO-1 immunoreactivity was observed in the ipsilateral cortex and striatum of WT and *IRG1*^−^^*/*^^−^ MCAO mice (Fig. 5C, left panel). Consistently, our results showed that the level of HO-1 immunoreactivity was significantly higher in the ipsilateral cortex and striatum of WT MCAO mice than those of *IRG1*^−^^*/*^^−^ MCAO mice. Importantly, we also observed that the number of HO-1-expressing Iba1^+^ cells was higher in the ischaemic brain of WT MCAO mice than that of *IRG1*^−^^*/*^^−^ MCAO mice (Fig. 5C, right panel).

To exam the effect of IRG1 deficiency on HO-1 expression *in vitro*, we compared the level of HO-1 expression in MG and MΦ generated from WT and *IRG1*^−^^*/*^^−^ mice following LPS stimulation. We first determined whether LPS induced IRG1 expression in MG and MΦ, and our results showed that IRG1 mRNA expression was upregulated at 5 h and declined at 8 h after LPS stimulation in both WT MG (Fig. 5D) and MΦ (Supplementary Fig. 5A). At protein levels, IRG1 was induced at 8 h and strongly upregulated at 16 and 24 h after LPS stimulation in both WT MG (Fig. 5E) and MΦ (Supplementary Fig. 5B). As expected, the protein expression of IRG1 was not observed in *IRG1*^−^^*/*^^−^ MG and MΦ after LPS stimulation (Fig. 5E and Supplementary Fig. 5B). We then compared the level of HO-1 expression in WT and *IRG1*^−^^*/*^^−^ MG and MΦ. We observed that the basal level of HO-1 mRNA expression was suppressed at 5 and 8 h post LPS treatment in both WT and *IRG1*^−^^*/*^^−^ MG. However, HO-1 was then upregulated in WT MG but not in *IRG1*^−^^*/*^^−^ MG at 24 h after LPS stimulation (Fig. 5F). Similar results were observed in MΦ in which HO-1 mRNA expression was highly upregulated in WT MΦ but not in *IRG1*^−^^*/*^^−^ MΦ after 24 h of LPS stimulation (Supplementary Fig. 5C). Collectively, our results demonstrate that ischaemic stroke induces IRG1 expression in MG, and deficiency in IRG1 results in repressed HO-1 expression in MG in the ischaemic brain *in vivo* and in MG stimulated with LPS *in vitro*.

### DMI induces HO-1 expression in MG and ameliorates brain injury in ischaemic stroke

**Figure 6:**
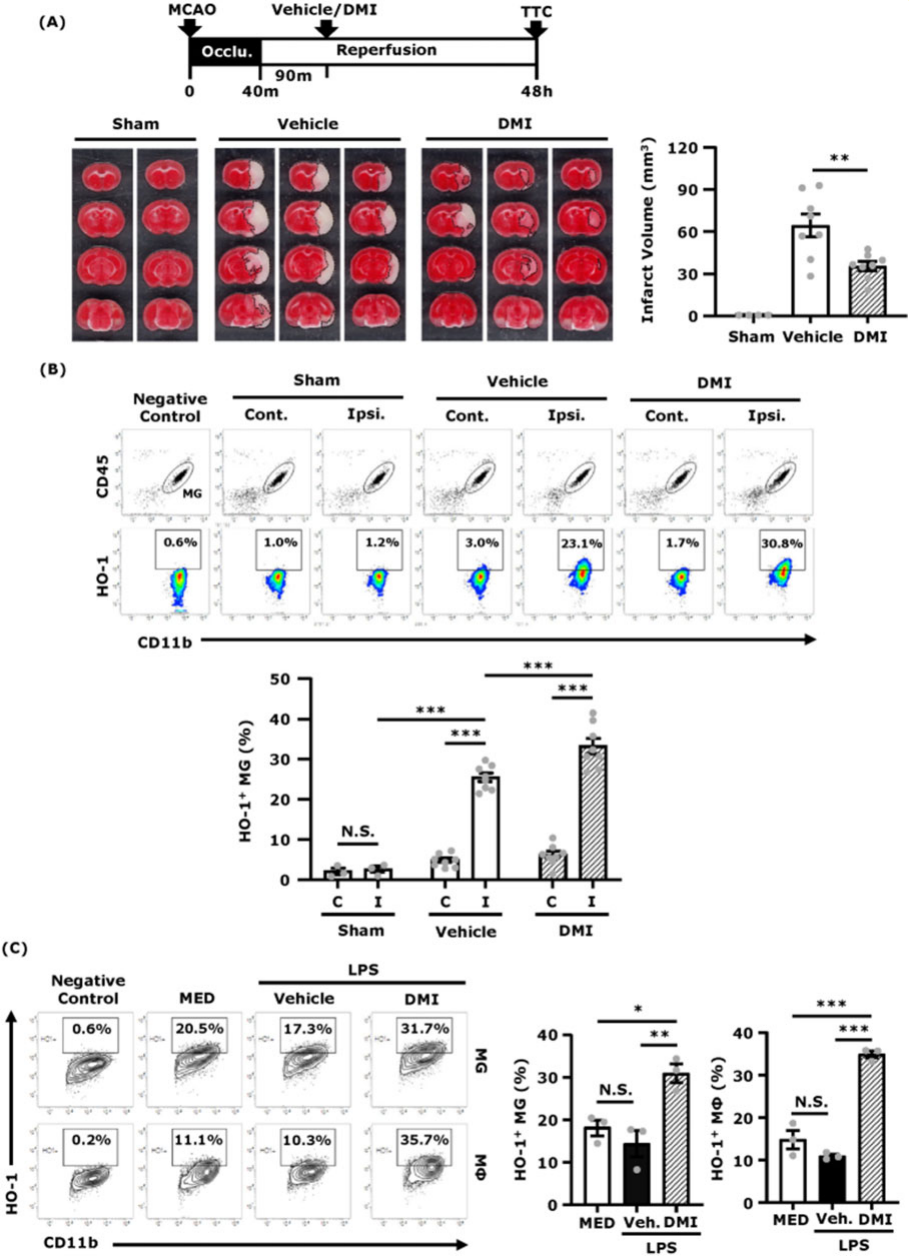
**DMI induces HO-1 expression in MG and ameliorates brain injury in ischaemic stroke.** C57BL/6 mice were subjected to sham or 40 min MCAO. The MCAO mice were then intraperitoneal administered with vehicle (DMSO) or DMI 500 mg/kg at 90 min post-reperfusion. (**A**) At 48 h post-injury, sham (*n* = 4 mice) and vehicle- and DMI-treated MCAO mice (*n* = 8 mice per group) were sacrificed, and the ischaemic brains were harvested and sliced (2 mm) followed by TTC staining. Two and three representative TTC-stained samples of sham and vehicle- and DMI-treated MCAO mice are shown. The infarct volumes of sham and vehicle- and DMI-treated MCAO mice were also measured. **P < 0.01 by one-way ANOVA. (**B**) At 15 h post-injury, the ischaemic brains were harvested from sham (*n* = 3 mice) and vehicle- and DMI-treated MCAO (*n* = 8 mice per group), and separated into the contralateral (Cont.; C) and ipsilateral (Ipsi.; I) hemispheres followed by mononuclear cell isolation. The isolated cells were then stained with antibodies of CD45 and CD11b followed by intracellular staining of primary HO-1 antibody and secondary Alexa Fluor 546 antibody. The cells were then subjected to flow cytometry analysis. Negative control (secondary antibody only) was used to determine CD45^int^CD11b^+^ MG positive for HO-1 expression. ***P < 0.001, N.S., no significant differences by two-way ANOVA. (**C**) Primary MG and MΦ were pretreated with vehicle (Veh.) or DMI 150 μM for 1 h followed by LPS 100 ng/ml stimulation. Six hours after LPS stimulation, cells were collected and subjected to surface staining of CD11b followed by intracellular staining of primary HO-1 antibody and secondary Alexa Fluor 546 antibody. The cells were then subjected to flow cytometry analysis. Negative control (secondary antibody only) was used to determine the positive expression of HO-1 in CD11b^+^ cells. The representative flow cytometry results of MG and MΦ are shown, and the frequency of HO-1^+^ MG and HO-1^+^ MΦ were measured (*n* = 3 biological replicates per group). *P < 0.05, **P < 0.01, ***P < 0.001, N.S., no significant differences by one-way ANOVA.

Since IRG1 catalyses the production of itaconate and itaconate has been shown to exert anti-inflammatory effects in several disease models,^12^^,^^24–29^ we investigated whether itaconate conferred protection against ischaemic brain injury. Mice were subjected to sham or MCAO followed by intraperitoneal administration of DMI, an itaconate derivative, and the level of cerebral infarct was then assessed at Day 2 post-injury. Our results showed that DMI-treated MCAO mice exhibited attenuated infarct volumes compared to vehicle-treated MCAO mice (DMI 35.4 ± 3.3 mm^3^ versus Vehicle 64.4 ± 8.1 mm^3^), suggesting DMI confers protection against ischaemic stroke (Fig. 6A).

Because DMI was reported to induce exogenous HO-1 expression^5^^,^^12^ and previous studies demonstrated that the induction of HO-1 offered protection against ischaemic stroke,^30^^,^^31^ we, therefore, evaluated whether DMI induced HO-1 expression in the ischaemic brain. Since MG were reported to be the main HO-1 producers in the CNS^32^ and we also observed HO-1 was highly expressed in MG in the ischaemic brain (Fig. 5A), we, therefore, assessed the level of HO-1 expression in MG in sham and vehicle- and DMI-treated MCAO mice. Our results showed that ischaemic stroke-induced HO-1 expression in MG compared to sham controls. Notably, DMI treatment further enhanced the frequency of HO-1-expressing MG compared to vehicle treatment (DMI 33.3 ± 1.9% versus Vehicle 25.5 ± 1.1%) in MCAO mice (Fig. 6B). Consistently, our *in vitro* studies also showed that DMI enhanced HO-1 expression in MG and MΦ stimulated with LPS (Fig. 6C). Taken altogether, our results demonstrate that DMI up-regulates HO-1 expression in MG, and that may contribute to its protective effects on the amelioration of brain injury in ischaemic stroke.

### DMI rescues IRG1 deficiency-exacerbated brain injury in ischaemic stroke

**Figure 7:**
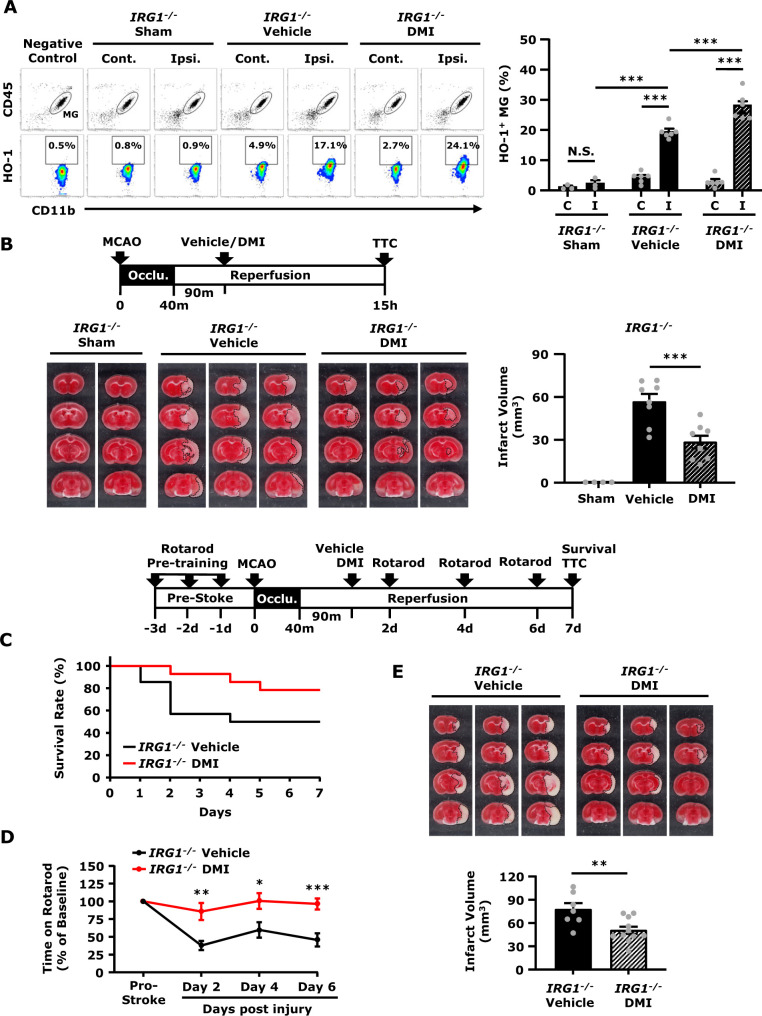
**DMI rescues IRG1 deficiency-exacerbated brain injury in ischaemic stroke.***IRG1*^−^^*/*^^−^ mice were subjected to sham or 40 min MCAO. *IRG1*^−^^*/*^^−^ MCAO mice were intraperitoneal administered with vehicle or DMI 500 mg/kg at 90 min post-reperfusion. (**A**) At 15 h post-injury, the ischaemic brains harvested from sham (*n* = 3 mice) and vehicle- and DMI-treated *IRG1*^−^^*/*^^−^ MCAO mice (*n* = 6 mice per group) were separated into the contralateral (Cont.; C) and ipsilateral (Ipsi.; I) hemispheres followed by mononuclear cell isolation. The isolated cells were then stained with antibodies of CD45 and CD11b followed by intracellular staining of primary HO-1 antibody and secondary Alexa Fluor 546 antibody. The cells were then subjected to flow cytometry analysis. Negative control (secondary antibody only) was used as a negative control to determine CD45^int^CD11b^+^ MG positive for HO-1 expression. ***P < 0.001, N.S., no significant differences by two-way ANOVA. (**B**) At 15 h post-injury, the ischaemic brains harvested from sham (*n* = 4 mice) and vehicle- and DMI-treated *IRG1*^−^^*/*^^−^ MCAO mice (*n* = 8 mice per group) were subjected to TTC staining. The representative TTC-stained samples of sham (*n* = 2 mice) and vehicle- and DMI-treated MCAO mice (*n* = 3 mice per group) are shown. The infarct volumes were also measured. ***P < 0.001 by one-way ANOVA. (**C–E**) *IRG1*^−^^*/*^^−^ MCAO mice were intraperitoneal administered with vehicle or DMI 500 mg/kg at 90 min post-reperfusion (*n* = 14 mice per group). Vehicle- and DMI-treated *IRG1*^−^^*/*^^−^ MCAO mice were subjected to (**C**) survival assay up to Day 7 post-injury and (**D**) rotarod tests at Day 2, 4 and 6 post-injury. *P < 0.05, **P < 0.01, ***P < 0.001 by unpaired *t*-*test*. (**E**) At Day 7 post-injury, the survived vehicle- (*n* = 7 mice) and DMI- (*n* = 11 mice) treated *IRG1*^−^^*/*^^−^ MCAO mice were sacrificed and subjected to TTC staining. Three representative TTC-stained samples are shown. The infarct volumes were also measured. **P < 0.01 by unpaired *t*-test.

Since repressed microglial HO-1 expression and exacerbated ischaemic brain injury were observed in *IRG1*^−^^*/*^^−^ MCAO mice, we speculated that HO-1 repression in the ischaemic brain would be responsible for exacerbated brain injury in *IRG1*^−^^*/*^^−^ MCAO mice. We, therefore, assessed whether the induction of exogenous HO-1 expression in MG by DMI could rescue IRG1 deficiency-exacerbated ischaemic brain injury. Indeed, our results showed that DMI treatment significantly increased the frequency of HO-1-expressing MG in the ischaemic brain compared to vehicle treatment in *IRG1*^−^^*/*^^−^ MCAO mice (DMI 28.3 ± 2.0% versus Vehicle 19.6 ± 0.9%) (Fig. 7A). Remarkedly, DMI treatment effectively ameliorated ischaemic brain injury in *IRG1*^−^^*/*^^−^ MCAO mice, as DMI-treated *IRG1*^−^^*/*^^−^ MCAO mice displayed attenuated infarct volumes compared to vehicle-treated *IRG1*^−^^*/*^^−^ MCAO mice (DMI 28.4 ± 4.5 mm^3^ versus Vehicle 56.7 ± 5.4 mm^3^) (Fig. 7B).

To further evaluate the long-term effect of DMI treatment on the amelioration of IRG1 deficiency-exacerbated ischaemic brain injury, *IRG1*^−^^*/*^^−^ mice were subjected to MCAO followed by the administration of vehicle or DMI, and the survival rate, rotarod test and infarct volume were assessed up to Day 7 post-injury. Our results showed that *IRG1*^−^^*/*^^−^ MCAO mice treated with DMI had a higher survival rate than those treated with the vehicle at Day 7 post-injury (DMI 78.6% versus Vehicle 50%) (Fig. 7C). Furthermore, DMI-treated *IRG1*^−^^*/*^^−^ MCAO mice exhibited a better outcome on rotarod tests compared to vehicle-treated *IRG1*^−^^*/*^^−^ MCAO mice (Fig. 7D). Finally, DMI-treated *IRG1*^−^^*/*^^−^ MCAO mice displayed smaller infarct volumes than vehicle-treated *IRG1*^−^^*/*^^−^ MCAO mice at Day 7 post-injury (DMI 50.7 ± 4.7 mm^3^ versus Vehicle 77.6 ± 8.0 mm^3^) (Fig. 7E). Taken altogether, our results demonstrate that DMI enhances HO-1 expression in MG and rescues IRG1 deficiency-exacerbated brain injury, leading to improved motor coordination, attenuated brain infarct and enhanced survival rate in *IRG1*^−^^*/*^^−^ MCAO mice.

## Discussion

IRG1, a mitochondrial enzyme, mediates the production of itaconate during inflammatory responses in myeloid lineage cells.^33^ Recent studies have revealed the unique function of the IRG1/itaconate axis in modulating inflammation and demonstrated the anti-inflammatory effects of itaconate on suppressing succinate dehydrogenase (SDH) activity, activating the Nrf2/HO-1 pathway and ameliorating disease severity in several animal disease models.^8^^,^^9^^,^^22^^,^^34–36^ Presently, the immunomodulatory effects of the IRG1/itaconate axis were mainly studied in the peripheral inflammatory immune responses. Recently, our group showed that DMI, an itaconate derivative, suppressed neuroinflammation and ameliorated disease severity in experimental autoimmune encephalomyelitis, a chronic CNS disease.^12^ However, whether IRG1/itaconate axis exerts effects on modulating acute CNS disease, such as ischaemic stroke, remains unexplored. Hence, in this study, we aimed to investigate the immunomodulatory effects of IRG1 on the amelioration of ischaemic brain injury and elucidate the molecular mechanisms underlying the protective effect of IRG1 in ischaemic stroke. We found that IRG1 was highly upregulated in the ischaemic brain after ischaemic stroke. Interestingly, we observed that mice deficient in IRG1 exhibited enhanced MG activation, aggravated BBB disruption and exacerbated brain injury after ischaemic stroke compared to their corresponding WT stroke controls. Mechanistic studies revealed that IRG1 deficiency-mediated HO-1 repression might be responsible for exacerbated ischaemic brain injury in *IRG1*^−^^*/*^^−^ MCAO mice. Importantly, the administration of DMI to compensate for the deficiency of IRG1/itaconate axis enhanced HO-1 expression in MG and ameliorated ischaemic brain injury in *IRG1*^−^^*/*^^−^ MCAO mice. Thus, our findings suggest that the induction of IRG1 following ischaemic stroke may serve as an endogenous protective mechanism to restrain brain injury through the induction of HO-1 in the ischaemic brain.

Neuroinflammation plays a pivotal role in brain injury following ischaemic stroke. MG activation and peripheral immune cell infiltration have been shown to contribute to the induction and aggravation of neuroinflammation in ischaemic stroke, respectively.^13^^,^^15^ Since we observed exacerbated ischaemic brain injury in *IRG1*^−^^*/*^^−^ MCAO mice, we speculated that IRG1 deficiency might promote neuroinflammation in ischaemic stroke. Indeed, when we analysed the ischaemic brain of WT and *IRG1*^−^^*/*^^−^ MCAO mice, we found increased CD68^+^ MG and CD86^+^ MG as well as elevated Iba1^+^ cells in the ischaemic brain of *IRG1*^−^^*/*^^−^ MCAO mice compared to WT MCAO mice, demonstrating IRG1 deficiency promotes MG activation in ischaemic stroke. Furthermore, we observed IRG1 deficiency led to augmented BBB disruption and increased immune cell infiltration of the CNS following ischaemic stroke. Importantly, our mechanistic studies revealed that deficiency in IRG1 resulted in enhanced MMP9 and MMP3 expression in the ischaemic brain, and that correlated with repressed HO-1 expression in the ischaemic brain of *IRG1*^−^^*/*^^−^ MCAO mice (Supplementary Fig. 2B). Since previous studies demonstrated that HO-1 exerted a modulatory effect on the production of MMP9 and MMP3,^37–40^ our results suggest that IRG1 deficiency-mediated HO-1 repression may contribute to enhanced MMP9 and MMP3 expression in the ischaemic brain of *IRG1*^−^^*/*^^−^ MCAO mice. Altogether, our results demonstrate that IRG1 exerts immunomodulatory effects on the attenuation of neuroinflammation through suppressing MG activation, lessening BBB disruption and diminishing peripheral immune cell infiltration of the CNS in ischaemic stroke.

In this study, we observed IRG1 was only upregulated in the CD11b^+^ but not CD11b^−^ cells isolated from the ipsilateral hemisphere of the ischaemic brain, suggesting MG are the main cell type expressing IRG1 in the ischaemic brain. These results are consistent with previous findings, showing IRG1 was expressed in MG but not in other CNS cells after LPS systemic challenge.^32^ The induction of IRG1 has been shown to increase endogenous itaconate production that subsequently activates Nrf2/HO-1 pathway in several disease models.^29^^,^^34–36^ Interestingly, a previous study reported that IRG1 could be induced by HO-1 inducers in combination with carbon monoxide to inhibit LPS-induced sepsis and pro-inflammatory cytokine production.^41^ These results suggest that the reciprocal effects may exist between IRG1 and HO-1 gene induction. In addition, when we analysed HO-1 expression in the isolated CD11b^+^ and CD11b^−^ cells, we found HO-1 was highly induced in the CD11b^+^ cells isolated from the ipsilateral hemisphere of WT MCAO mice compared to those of *IRG1*^−^^*/*^^−^ MCAO mice, suggesting IRG1 promotes HO-1 expression in the ischaemic brain. Notably, HO-1 upregulation was also observed in the CD11b^+^ cells isolated from the ipsilateral hemisphere of *IRG1*^−^^*/*^^−^ MCAO mice compared to those isolated from the contralateral hemisphere of *IRG1*^−^^*/*^^−^ MCAO mice, indicating IRG1-independent mechanism of inducing HO-1 expression exists in MG after ischaemic strokes, such as the direct activation of Nrf2/HO-1 pathway by ischaemia-induced oxidative stress. Although we observed both CD11b^+^ and CD11b^−^ cells isolated from the ipsilateral hemisphere of WT MCAO mice exhibited increased HO-1 expression, HO-1 expression was significantly higher in CD11b^+^ than CD11b^−^ cells, suggesting ischaemic stroke induces HO-1 expression mainly in MG but also in other CNS cells. Indeed, a previous study reported that MG expressed a high level of HO-1, and brain endothelial cells, astrocytes and oligodendrocytes expressed a moderate to low level of HO-1.^32^ Finally, previous studies showed that MG deletion exacerbated brain injury after ischaemic stroke.^42^^,^^43^ These results highly correlate with our findings, demonstrating that ischaemic stroke induces IRG1 expression in MG and deficiency in IRG1 results in exacerbated ischaemic brain injury. However, studies using IRG1 conditional knockout specifically in MG (*IRG1*^fl/fl^:*CX3CR1*^CreERT2^) would be warranted to confirm the protective role of IRG1-expressing MG in ischaemic stroke.

Previous studies showed that the induction of HO-1 exerted potent protection against ischaemic stroke.^15^^,^^44–47^ In addition, a study showed that HO-1 plays an essential role in ischaemic preconditioning-induced protection against brain ischaemia, as ischaemic preconditioning failed to protect HO-1 deficient mice against permanent ischaemic brain injury.^31^ In this study, we found that both IRG1 and HO-1 were highly upregulated in the ischaemic brain of WT MCAO mice. However, ischaemia-induced HO-1 upregulation was repressed in the ischaemic brain of *IRG1*^−^^*/*^^−^ MCAO mice, and that correlated with exacerbated ischaemic brain injury observed in *IRG1*^−^^*/*^^−^ MCAO mice. These findings strongly imply the protective effect of HO-1 in ischaemic stroke. We also verified the role of IRG1 in promoting HO-1 expression *in vitro*. We found MG and MΦ generated from *IRG1*^−^^*/*^^−^ mice failed to optimally upregulate HO-1 expression following LPS stimulation compared to MG and MΦ generated from WT mice. Finally, we confirmed the protective effect of HO-1 on the amelioration of ischaemic brain injury by administering *IRG1*^−^^*/*^^−^ MCAO mice with DMI. We observed DMI enhanced HO-1 upregulation in MG and attenuated infarct volumes during the acute phase (Day 1 post-injury), and lessened ischaemic brain injury, improved motor coordination and enhanced survival during the sub-acute phase (Day 7 post-injury) in *IRG1*^−^^*/*^^−^ MCAO mice. Collectively, our findings demonstrate that the induction of IRG1 following ischaemic stroke promotes HO-1 expression that may subsequently restrain ischaemic brain injury.

Nevertheless, an additional molecular mechanism by which the IRG1/itaconate axis confers protection against ischaemic stroke may exist. During respiration with normal oxygen consumption, SDH catalyses succinate to fumarate by oxidation in the tricarboxylic acid cycle. However, during ischaemia SDH works reversely, leading to succinate accumulation. Indeed, studies have reported that a high level of succinate could be detected in the ischaemic tissues of the heart, liver, kidney and brain.^48^ The accumulated succinate can then be rapidly oxidized following reperfusion, resulting in increased mitochondrial reactive oxygen species production. Notably, the inhibition of SDH activity was reported to confer protection against ischaemic injury.^48^^,^^49^ As IRG1/itaconate axis exerts an effect on the suppression of SDH activity,^8^^,^^50^ further studies would be required to investigate whether IRG1 deficiency-exacerbated ischaemic brain injury may be partly due to unregulated SDH activity that leads to increased succinate accumulation and elevated reactive oxygen species production in the ischaemic brain.

In summary, we demonstrate that the induction of IRG1 following ischaemic stroke may serve as an endogenous protective mechanism to restrain ischaemic brain injury. We found that deficiency in IRG1 resulted in exacerbated brain injury in ischaemic stroke in which *IRG1*^−^^*/*^^−^ stroke animals exhibited elevated MG activation, aggravated BBB disruption and enlarged cerebral infarct in the ischaemic brain. Moreover, we showed that ischaemic stroke induced IRG1 expression in MG and IRG1 promoted microglial HO-1 expression that might subsequently modulate ischaemic brain injury. Finally, we showed that IRG1 deficiency-exacerbated ischaemic brain injury could be rescued by DMI via the induction of exogenous HO-1 expression in MG. Thus, our study reveals a novel function of IRG1 in modulating brain injury following ischaemic stroke, and that suggests targeting IRG1 and/or IRG1-mediated signalling pathways may represent a novel therapeutic strategy for the treatment of ischaemic stroke.

## Supplementary material

Supplementary material is available at *Brain Communications* online.

## Funding

This work was supported by Indiana University Startup Fund and in part by the National Institutes of Health (R01NS102449) to J.-H.Y.

## Competing interests

The authors declare no conflict of interests.

## Supplementary Material

fcab187_Supplementary_Data

## Abbreviations

BBB =: blood–brain barrier
CBF =: cerebral blood flow
DMI =: dimethyl itaconate
HO-1 =: heme oxygenase-1
IHC =: immunohistochemistry
IRG1 =: immunoresponsive gene 1
LPS =: lipopolysaccharide
MCAO =: middle cerebral artery occlusion
MG =: microglia
MΦ =: macrophages
Nrf2 =: nuclear factor erythroid 2-related factor 2
RT =: room temperature
TTC =: triphenyltetrazolium chloride
WT =: wild type
