# Supplementary material for: Immunoresponsive gene 1 modulates the severity of brain injury in cerebral ischaemia
Source: Brain Commun. 2021 Aug 19;3(3):fcab187. doi: 10.1093/braincomms/fcab187 (PMC8453405; doi:10.1093/braincomms/fcab187)
Supplement: fcab187_Supplementary_Data [file fcab187_Supplementary_Data.pdf]

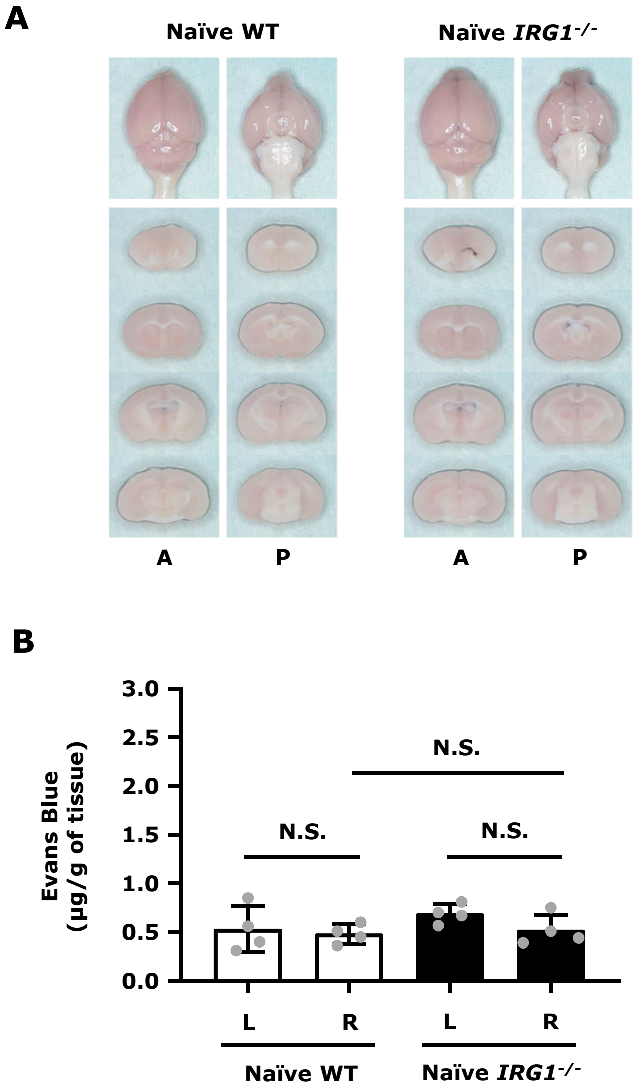

Supplemental Figures 1 IRG1 deficiency does not alter blood-brain barrier integrity. Naïve WT and *IRG1*$^{-/-}$ mice were intravenously administrated with Evans blue. One hour post-administration, brains were harvested followed by imaging and sectioning. (A) The representative images of naïve WT and *IRG1*$^{-/-}$ mice are shown. A: anterior surface; P: posterior surface. (B) The Evans blue leakages in the left (L) and right (R) hemispheres were measured (n=4 mice per group). N.S., no significant differences by two-way ANOVA.

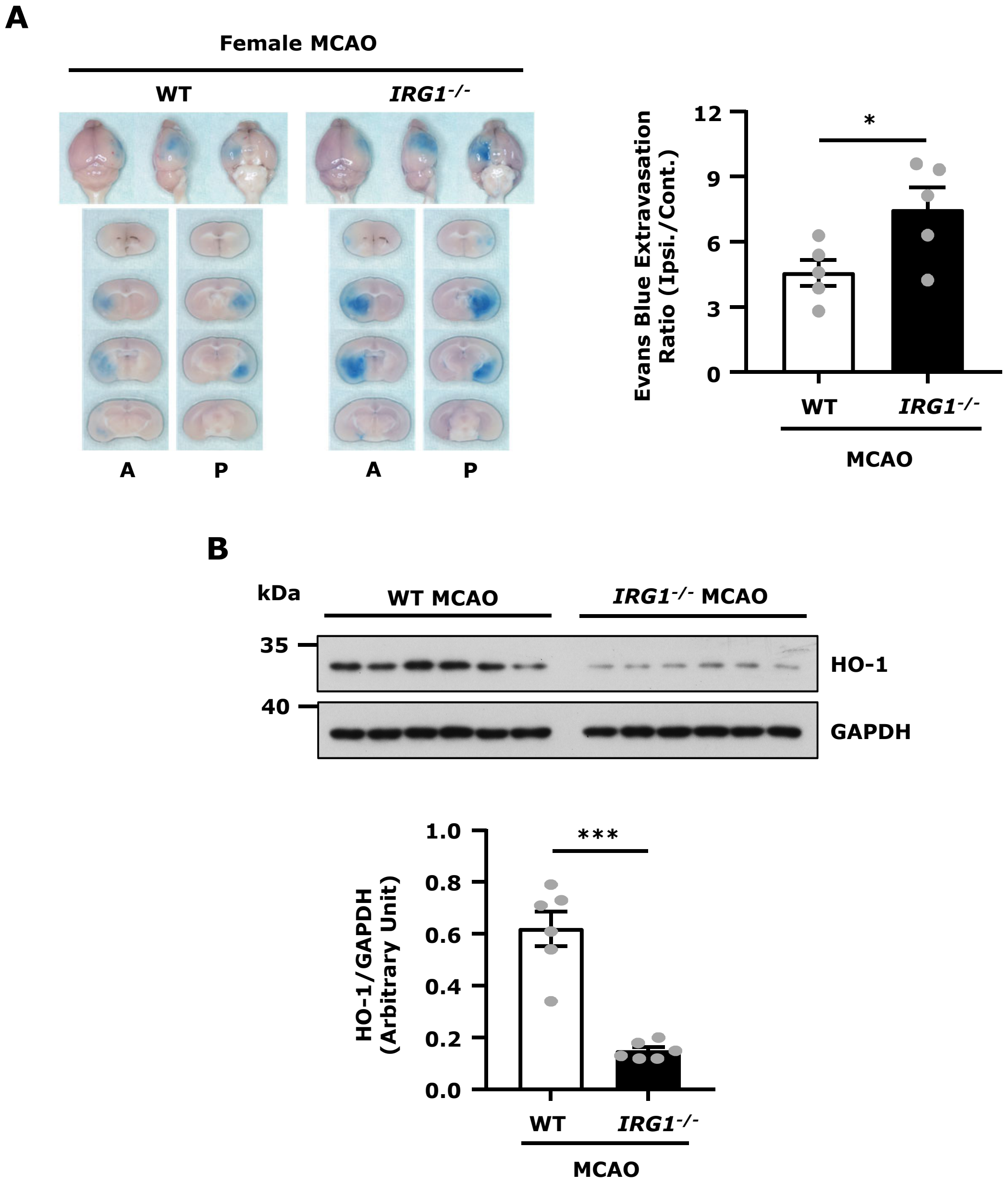

Supplemental Figure 2 Deficiency in IRG1 results in aggravated BBB disruption and repressed HO-1 expression in the ischemic brain. (A) Female WT and *IRG1*$^{-/-}$ mice were subjected to 3 hours MCAO followed by 3.5 hours reperfusion (n=5 mice per group). One hour prior scarification, mice were intravenously administered with Evans blue. At 6.5 hours post-injury, the ischemic brains were then harvested and subjected to sectioning and imaging, and the Evans blue leakage in the contralateral and ipsilateral hemispheres was then quantified. The representative ischemic brain images of WT and *IRG1*$^{-/-}$ MCAO mice are shown. A: anterior surface; P: posterior surface. The Evans blue extravasation ratio of ipsilateral (Ipsi.) hemisphere / contralateral (Cont.) hemisphere was also determined. *$p<0.05$ by unpaired t test. (B) Male WT and *IRG1*$^{-/-}$ mice were subjected to 3 hours MCAO, and the ipsilateral hemispheres were then harvested at 3.5 hours post-reperfusion followed by western blot analysis for HO-1 expression (n=6 mice per group). The level of HO-1 expression was also quantified. ***$p<0.001$ by unpaired t test.

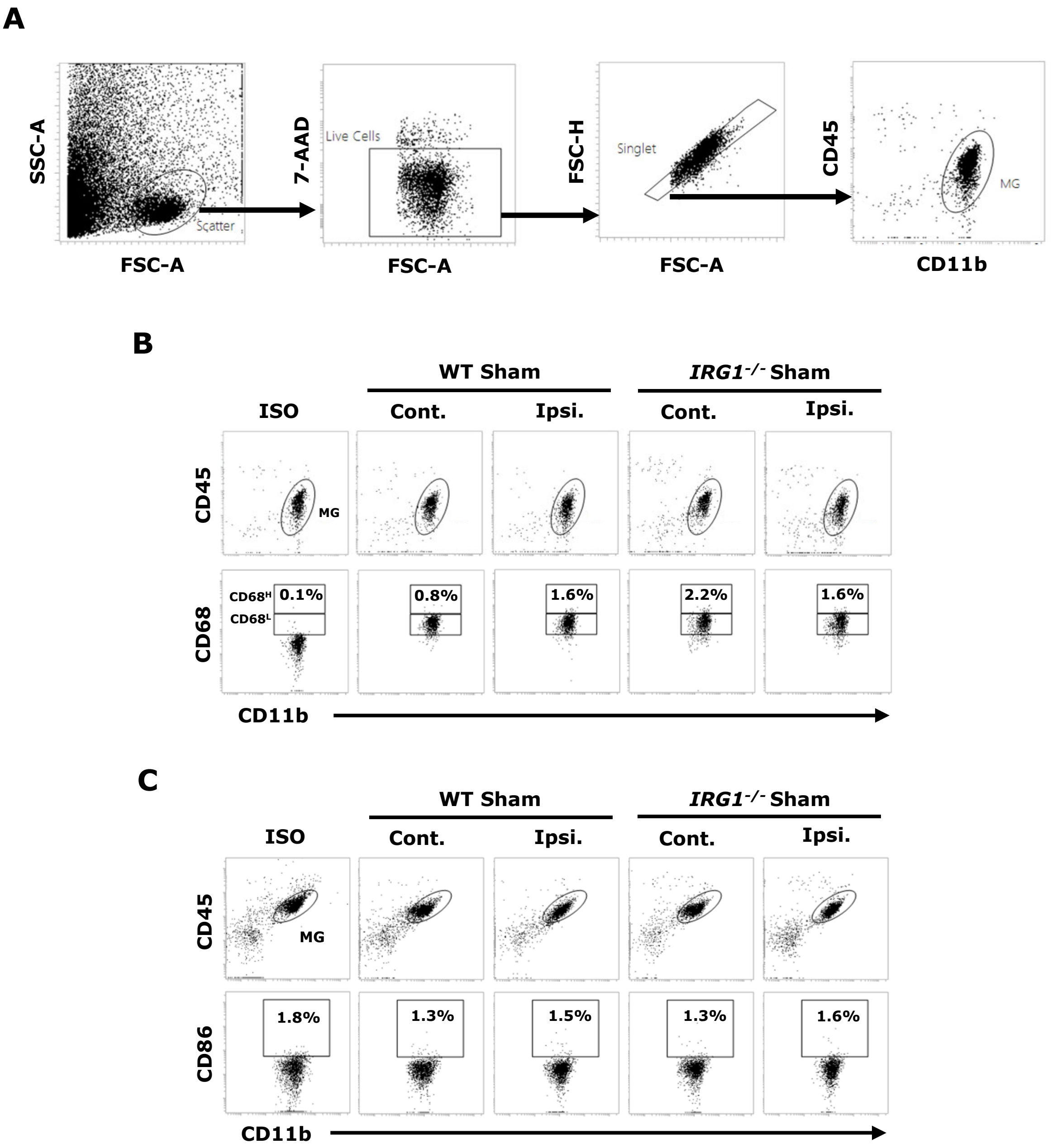

Supplemental Figure 3 The expression of CD68 and CD86 in MG in WT and *IRG1*$^{-/-}$ sham mice. WT and *IRG1*$^{-/-}$ mice were subjected to sham surgery, and mice were sacrificed at 15 hours post-injury. The ischemic brains were then harvested and subjected to mononuclear cell isolation followed by flow cytometry analysis for CD68 or CD86 expression in MG. (A) Cells were first gated on mononuclear cells (SSC-A vs. FSC-A) and then on 7-AAD negative cells. 7-AAD^{-} live cells were then gated on singlets (FSC-H vs. FSC-A) followed by gating on CD45^{int}CD11b^{+} MG. (B) The representative results of CD68^{L} and CD68^{H} MG in the contralateral (Cont.) and ipsilateral (Ipsi.) hemispheres of WT and *IRG1*$^{-/-}$ sham mice are shown. (C) The representative results of CD86 expression in MG in the contralateral (Cont.) and ipsilateral (Ipsi.) hemispheres of WT and *IRG1*$^{-/-}$ sham mice are shown.

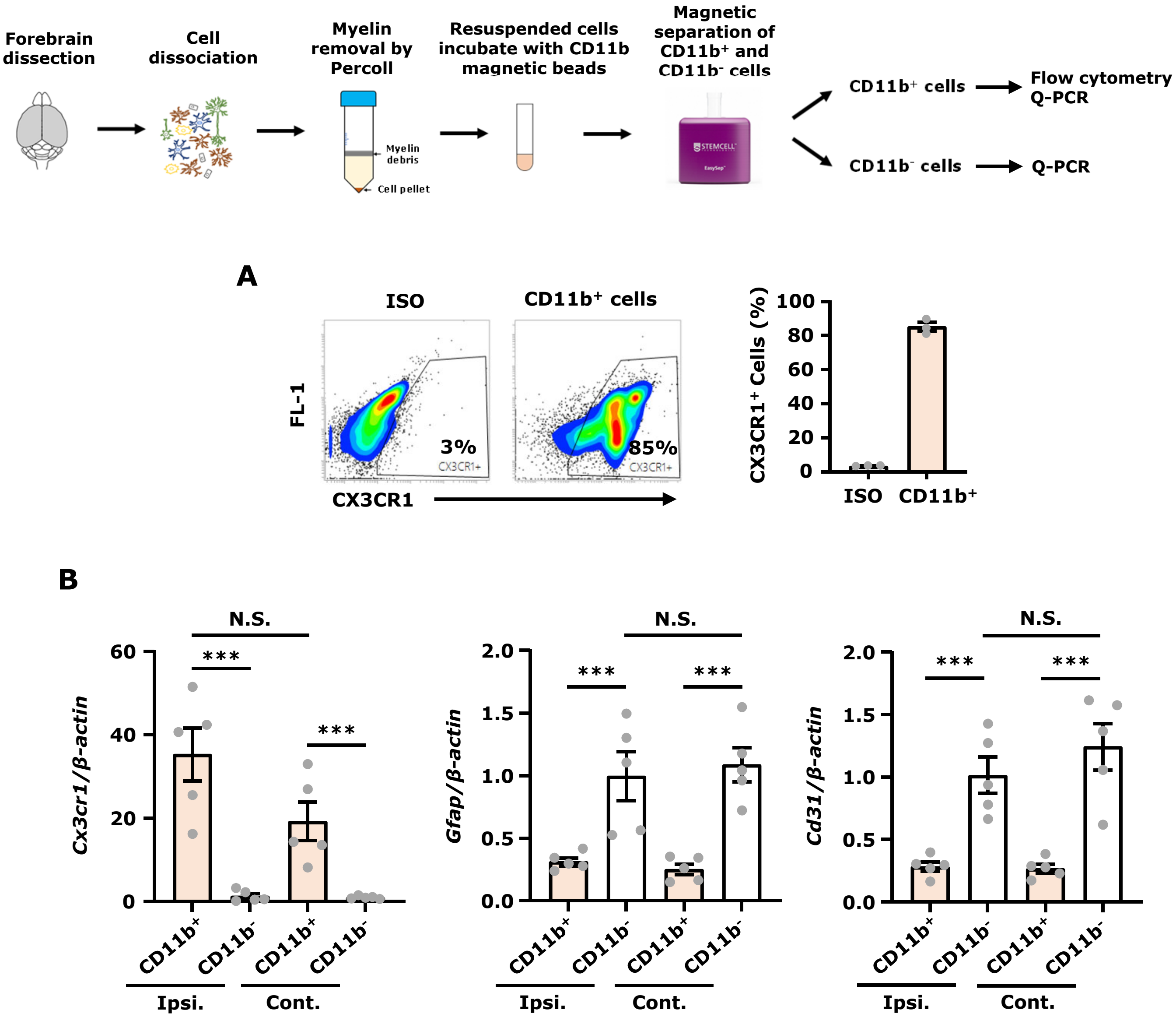

Supplemental Figure 4 CD11b isolation of the CNS cells followed by flow cytometery and Q-PCR analysis. (A) The brain tissues harvested from C57BL/6 mice were homogenized followed by CD11b isolation. The isolated $CD11b^+$ cells were then subjected to flow cytometery analysis to measure the frequency of $CX3CR1^+$ cells (n=3 mice). (B) C57BL/6 mice were subjected to 40 minutes MCAO. The ipsilateral (Ipsi.) and contralateral (Cont.) hemispheres were harvested at 6 hours post-reperfusion. Two ipsilateral or contralateral hemispheres were pooled and subjected to CD11b isolation. The $CD11b^+$ and $CD11b^-$ cells were then collected for RNA extraction followed by Q-PCR analysis for CX3CR1, GFAP, and CD31 expression (n= 5 per group, each n represents pooled two brain samples). ****p<0.001,* N.S., no significant differences by two-way ANOVA.

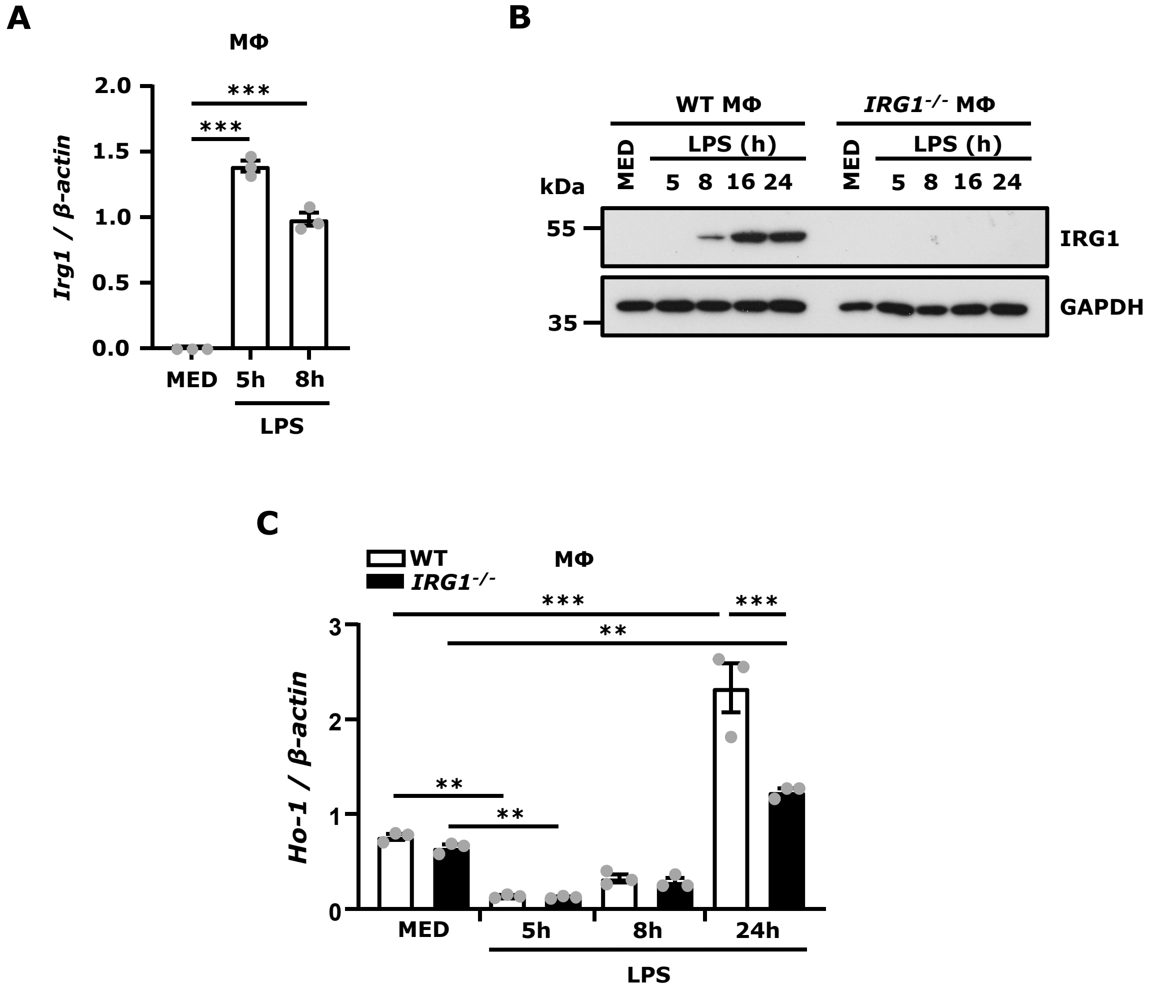

Supplemental Figure 5 MΦ deficient in IRG1 exhibit reduced HO-1 expression following LPS stimulation. (A) Primary MΦ generated from WT mice were stimulated with LPS 100 ng/ml for 5 or 8 hours, and cells were then harvested and subjected to Q-PCR analysis for IRG1 expression (n=3 technique replicates per group). ****p<0.001* by one-way ANOVA. (B) Primary MΦ generated from WT and *IRG1*$^{-/-}$ mice were stimulated with LPS 100 ng/ml for a time course followed by western blot analysis for IRG1 expression. (C) Primary WT and *IRG1*$^{-/-}$ MΦ were treated with LPS 100 ng/ml for a time course, and cells were then harvested followed by Q-PCR analysis for HO-1 expression (n=3 technique replicates per group). ***p<0.01*, ****p<0.001* by two-way ANOVA. The results of A to C represent 3-4 independent biological replicates.

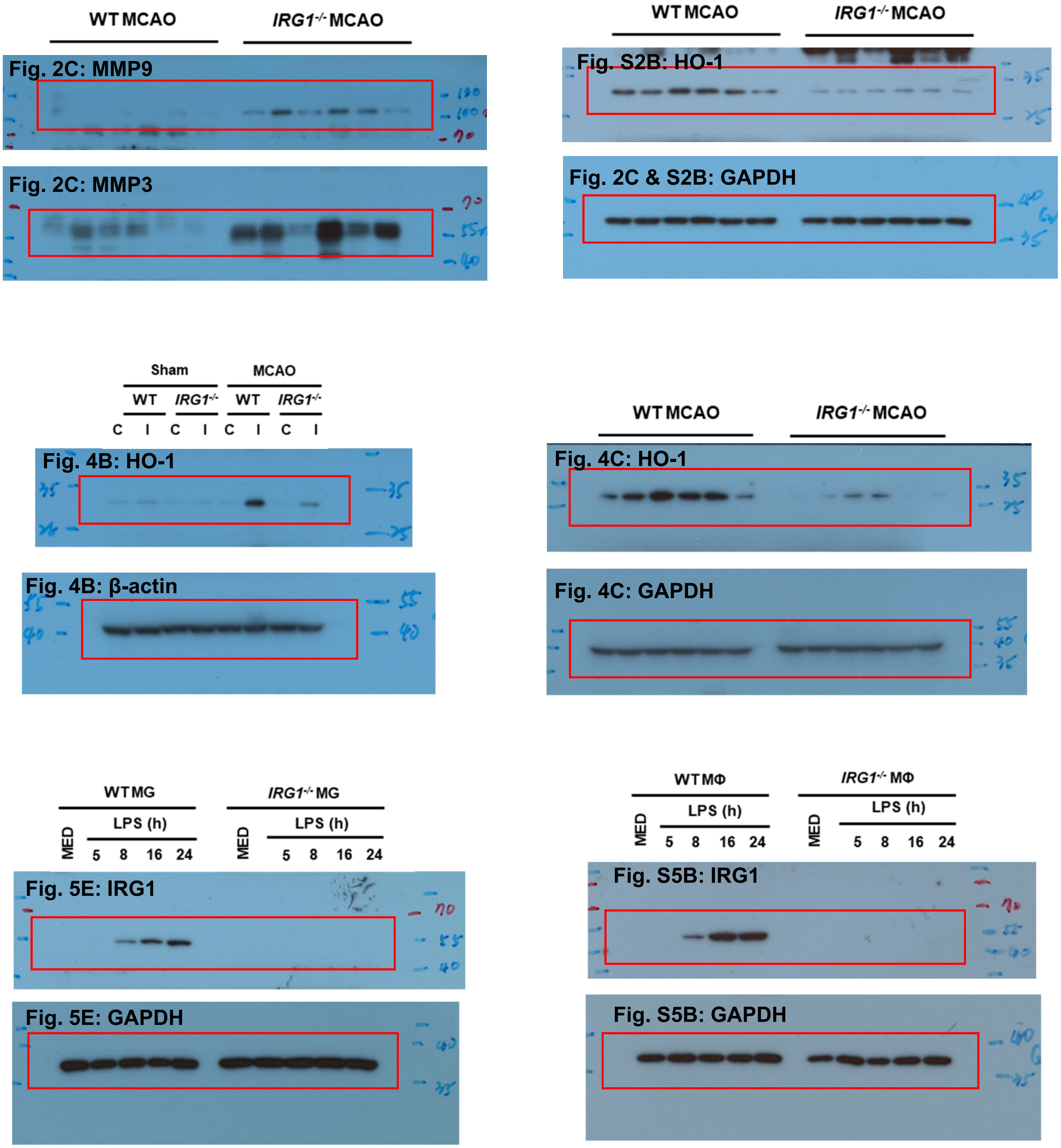

Supplemental Figure 6 Original western blot images. The original non-cropped X-ray film images of Fig. 2C, 4B, 4C, 5E, S2B, and S5B.

Supplemental Table 1:

Physiological parameters of mice during MCAO in Fig.1C.

			During MCAO		
Strain	Group	Weight (g)	Temperature (°C)	Oxygen Saturation (%)	Cerebral Blood Flow (% reduction)
C57BL/6 Wild type	Sham (n=4)	24.2 ± 0.2	36.6 ± 0.1	96.5 ± 0.4	--
	Control (n=10)	27.4 ± 0.4	36.7 ± 0.1	96.6 ± 0.5	89.3 ± 1.0
IRG1$^{-/-}$	Sham (n=4)	24.8 ± 0.8	36.6 ± 0.1	96.6 ± 0.4	--
	Control (n=10)	27.4 ± 0.4	36.8 ± 0.1	96.2 ± 0.5	87.8 ± 1.1

Data are shown as mean ± SEM.

Supplemental Table 2:

Physiological parameters of mice during MCAO in Fig.1E.

			During MCAO		
Strain	Group	Weight (g)	Temperature (°C)	Oxygen Saturation (%)	Cerebral Blood Flow (% reduction)
C57BL/6 Wild type	Control (n=7)	29.0 ± 1.5	36.6 ± 0.1	96.8 ± 0.2	93.5 ± 0.6
IRG1$^{-/-}$	Control (n=7)	27.0 ± 0.8	36.8 ± 0.1	95.9 ± 0.7	87.7 ± 1.5

Data are shown as mean ± SEM.

Supplemental Table 3:

Physiological parameters of mice during MCAO in Fig. 6A.

			During MCAO		
**Strain**	**Group**	**Weight (g)**	**Temperature (°C)**	**Oxygen Saturation (%)**	**Cerebral Blood Flow (% reduction)**
**C57BL/6 Wild type**	**Sham (n=4)**	**26.1 ± 0.2**	**36.8 ± 0.1**	**97.2 ± 0.3**	**--**
**C57BL/6 Wild type**	**Vehicle (n=8)**	**26.3 ± 0.4**	**36.8 ± 0.1**	**97.0 ± 0.4**	**91.5 ± 0.9**
**C57BL/6 Wild type**	**DMI (n=8)**	**25.6 ± 0.4**	**36.8 ± 0.1**	**96.8 ± 0.3**	**91.4 ± 1.2**

Data are shown as mean ± SEM.

Supplemental Table 4:

Physiological parameters of mice during MCAO in Fig. 7B.

			During MCAO		
**Strain**	**Group**	**Weight (g)**	**Temperature (°C)**	**Oxygen Saturation (%)**	**Cerebral Blood Flow (% reduction)**
IRG1$^{-/-}$	**Sham (n=4)**	**25.4 ± 0.5**	**36.6 ± 0.1**	**96.4 ± 0.6**	**--**
IRG1$^{-/-}$	**Vehicle (n=8)**	**26.2 ± 1.0**	**36.8 ± 0.1**	**96.2 ± 0.5**	**91.4 ± 0.8**
IRG1$^{-/-}$	**DMI (n=8)**	**24.1 ± 0.5**	**36.7 ± 0.1**	**96.8 ± 0.5**	**88.8 ± 1.1**

Data are shown as mean ± SEM.

Supplemental Table 5:

Physiological parameters of mice during MCAO in Fig. 7E.

			During MCAO		
Strain	Group	Weight (g)	Temperature (°C)	Oxygen Saturation (%)	Cerebral Blood Flow (% reduction)
IRG1$^{-/-}$	Vehicle (n=7)	26.2 ± 0.7	36.7 ± 0.1	97.2 ± 0.3	91.6 ± 1.2
IRG1$^{-/-}$	DMI (n=11)	26.3 ± 0.4	36.7 ± 0.1	97.2 ± 0.3	87.4 ± 2.4

Data are shown as mean ± SEM.
